# Probing the Limits of Reactant Concentration and Volume in Primitive Polyphenyllactate Synthesis and Microdroplet Assembly Processes

**DOI:** 10.1021/acsbiomedchemau.4c00082

**Published:** 2025-01-09

**Authors:** Mahendran Sithamparam, Rehana Afrin, Navaniswaran Tharumen, Ming-Jing He, Chen Chen, Ruiqin Yi, Po-Hsiang Wang, Tony Z. Jia, Kuhan Chandru

**Affiliations:** † Space Science Center (ANGKASA), Institute of Climate Change, National University of Malaysia, Selangor 43650, Malaysia; ‡ Earth-Life Science Institute, Institute of Future Science, Institute of Science Tokyo, 2-12-1-IE-1 Ookayama, Meguro-ku, Tokyo 152-8550, Japan; § Department of Chemical Engineering and Materials Engineering, National Central University, No. 300, Zhongda Rd., Zhongli District, Taoyuan 32001, Taiwan (R.O.C.); ∥ Biofunctional Catalyst Research Team, RIKEN Center for Sustainable Resource Science (CSRS), 2-1 Hirosawa, Wako, Saitama 351-0198, Japan; ⊥ State Key Laboratory of Isotope Geochemistry and CAS Center for Excellence in Deep Earth Science, Guangzhou Institute of Geochemistry, Chinese Academy of Sciences, Guangzhou 510640, China; # Graduate Institute of Environmental Engineering, National Central University, No. 300, Zhongda Road, Zhongli District, Taoyuan City 320, Taiwan; 7 Blue Marble Space Institute of Science, 600 first Ave, Floor 1, Seattle, Washington 98104, United States; 8 Polymer Research Center (PORCE), Faculty of Science and Technology, National University of Malaysia, Selangor 43600 Malaysia; 9 Institute of Physical Chemistry, CENIDE, University of Duisburg-Essen, 45141 Essen, Germany

**Keywords:** phase separation, origin of life, protocells, polyesters, prebiotic chemistry, membraneless compartments

## Abstract

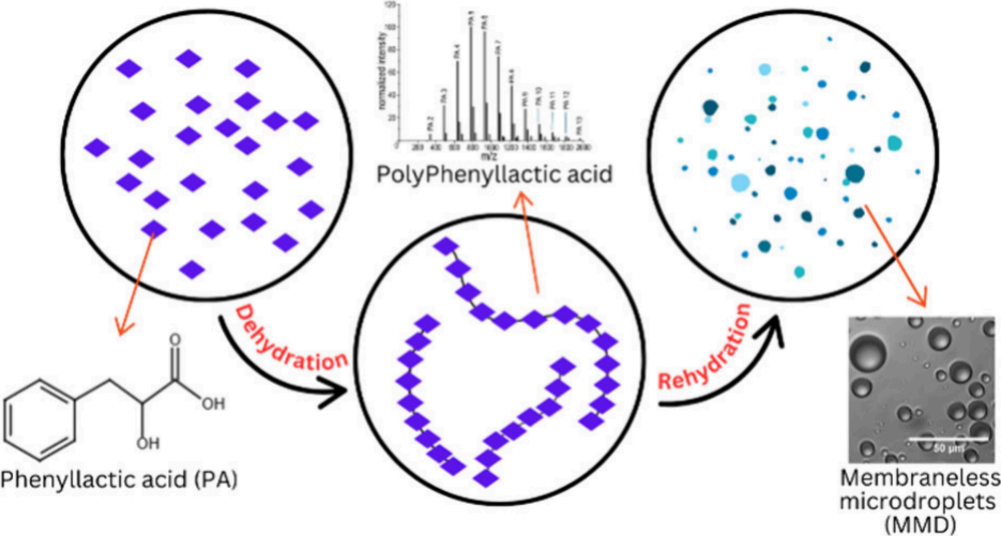

Polyester microdroplets have been investigated as primitive protocell models that can exhibit relevant primitive functions such as biomolecule segregation, coalescence, and salt uptake. Such microdroplets assemble after dehydration synthesis of alpha-hydroxy acid (αHA) monomers, commonly available on early Earth, via heating at mild temperatures, followed by rehydration in aqueous media. αHAs, in particular, are also ubiquitous in biology, participating in a variety of biochemical processes such as metabolism, suggesting the possible strong link between primitive and modern αHA-based processes. Although some primitive αHA polymerization conditions have been probed previously, including monomer chirality and reaction temperature, relevant factors pertaining to early Earth’s local environmental conditions that would likely affect primitive αHA polymerization are yet to be fully investigated. Hence, probing the entire breadth of possible conditions that could promote primitive αHA polymerization is required to understand the plausibility of polyester microdroplet assembly on early Earth at the origin of life. In particular, there are numerous aqueous environments available on early Earth that could have resulted in varying volumes and concentrations of αHA accumulation, which would have affected subsequent αHA polymerization reactions. Similarly, there were likely varying levels of salt in the various aqueous prebiotic solutions, such as in the ocean, lakes, and small pools, that may have affected primitive reactions. Here, we probe the limits of the dehydration synthesis and subsequent membraneless microdroplet (MMD) assembly of phenyllactic acid (PA), a well-studied αHA relevant to both biology and prebiotic chemistry, with respect to reactant concentration and volume and salinity through mass spectrometry- and microscopy-based observations. Our study showed that polymerization and subsequent microdroplet assembly of PA appear robust even at low reactant concentrations, smaller volumes, and higher salinities than those previously tested. This indicates that PA-polyester and its microdroplets are very much viable under a wide variety of conditions, thus more likely participating in prebiotic chemistries at the origins of life.

## Introduction

Abiogenesis describes the chemical evolution of life on early Earth, i.e., the origins of life (OoL), and involves the formation of simple organic molecules, their polymerization and self-assembly into complex molecules, the emergence of protocells,^1−3^ and the development of robust Darwinian evolution before or at the onset of the last universal common ancestor (LUCA).^4^ In particular, the synthesis of the primitive chemicals potentially leading to life could have taken place in various settings or by various geological processes on early Earth, including Miller–Urey chemistry,^5^ hydrothermal vents,^6,7^ shallow pools and lakes,^8,9^ panspermia delivery,^10^ or wet–dry cycles,^11,12^ just to name a few, and likely facilitated possible pathways toward several OoL hypotheses, i.e., the lipid-first world,^13^ the metabolism-first world,^14^ the RNA world,^15^*etc*. These primitive environments or processes, combined with energy (i.e., lightning strikes;^16^ light, UV-rays, and heat from the young sun;^17^ energy from radioactive elements;^18^ ionizing radiation;^19^*etc*.) could have facilitated the formation of many simple biomolecules on early Earth such as amino acids,^20^ lipids,^21^ nucleotides,^22^ simple sugars,^23^ or even phosphorus-containing compounds,^24^ which could have exhibited important functions or contributions to possibly kick-start the OoL.

However, it is important to recognize that the OoL is not necessarily strictly bound to the canonical biomolecules (i.e., lipids, amino acids, *etc.*). Other prebiotically available organic molecules could have also played equally essential roles during the emergence of life.^25,26^ In particular, we speculate that the properties of α-hydroxy acids (αHAs) lead this category of molecules to be potential key compounds at the OoL due to their active participation of αHAs in chemistries ranging from the prebiotic world all the way to modern biology. For example, citric acid (CA) and malic acid, both αHAs, serve as intermediates in the Krebs cycle.^27−29^ Ribosomes, traditionally known for synthesizing proteins, have also been shown to polymerize various αHAs, including lactic acid (LA) and phenyllactic acid (PA), into polyesters, a process that can be directed by mRNA through genetic-code reprogramming.^30^ Apart from αHA involvement in biology, αHAs are also essential in biotechnology and biomedicine.^31−38^

Recent studies also highlight the possible contribution of αHAs to the OoL. In particular, αHAs would have likely been readily available on early Earth as a result of prebiotic synthesis through various prebiotically plausible processes, such as in meteorites,^39^ via Miller–Urey spark discharge,^5^ as a product of atmospheric photochemical reactions,^40^ and in volcanic conditions.^41^ Once produced, these αHAs could have played a role in forming larger organic molecules on early Earth, which can be vital components of a primitive biological system. For example, the reaction of prebiotically available alpha-amino acids (αAAs) with αHAs could lead to the formation of depsipeptides, polymers that contain both ester bonds and amide bonds, which may have been the precursors to peptides^42−45^ and subsequently fostered protein-nucleic acid coevolution via their stabilizing effects on early nucleic acids.^46^ In fact, αHAs themselves possess the ability to polymerize through dehydration synthesis over short periods (geologically speaking) at mild temperatures (60–100 °C),^47^ a process that could have facilitated cyclical processes as part of terrestrial wet–dry cycles. In particular, after dehydration synthesis of αHAs, polymerization products, i.e., polyesters, exhibit a gel-like form.^47^ When these polyester gels are rehydrated in an aqueous medium, membraneless microdroplets (MMDs) spontaneously assembled.^48^ These MMDs likely assemble via a segregated phase separation process, where the extremely apolar interior is incompatible with that of the polar aqueous solvent. Previous work showing a negative MMD surface charge also suggests perhaps the free terminal carboxyl group plays a role in long-term droplet viability and structure, as neutralization of the surface charge by salt addition results in rapid droplet coalescence.^49^ There are many phase-separated compartments that serve as organelles in a cell having essential structural and catalytic functions, while aberrant intracellular phase separation leads to neurodegenerative diseases in modern biology.^50,51^ Likewise, primitive MMDs can perform prebiotically relevant functions such as selective segregation of small organics,^48^ functional RNA,^52^ and salts,^49^ as well as growth and coalescence.^49^ Thus, these possible primitive functions suggest the potential for polyester MMDs to have played a crucial role on early Earth as protocells, precursors of modern cells. Specifically, the MMDs are speculated to potentially provide a venue for prebiotic chemical reactions by encapsulating and exchanging essential primitive metabolic, genetic, and catalytic components.^25,53^ Compartmentalizing molecules over extended periods would have been necessary for the accumulation and concentration of prebiotic material, which could help the reactants reach the minimum levels needed for reactions to occur and facilitate the formation of more complex primitive structures or entities.^10,54,55^

Hence, research on emergent properties of primitive polyesters and MMDs could be crucial to understanding the emergence of life on Earth and/or for seeking life outside of Earth that may not necessarily use biomolecules as we know them.^25,26^ Nevertheless, further characterization of polymerization conditions must be explored before fully understanding the roles and plausibility of polyester MMDs at the OoL.

We have previously shown the effects of differing reaction temperatures and monomer chiralities in dehydration synthesis reactions of LA and PA to form polyesters and MMDs.^56^ However, this work along with all of our previous studies were conducted under controlled laboratory conditions^47−49,52,56,57^ (shown for comparison in Table S1, Supporting Information), and serves simply as a proof of concept and does not necessarily reflect actual prebiotic conditions. Therefore, investigating the environmental and geological factors that influence these processes on early Earth is crucial. By probing these limits, we can better understand the factors that likely influence these reactions. For example, one important factor is the availability of reactants on early Earth. Reactions were often inefficient due to low reactant concentrations or reactivities, leading to lower product yields to participate in downstream chemical reactions. Similarly, aqueous availability and reaction volume in different environments on early Earth would have also been inconsistent, where environmental processes, such as evaporation or precipitation, could affect reaction medium volume. Finally, salinity across bodies of water on both modern and primitive Earth is highly variable, and extreme salinities could result from the evaporation of even just slightly saline solutions.

Consequently, in this study, we aim to demonstrate the range of concentration, volume, and salts conditions under which αHA polymerization and polyester MMD remain plausible. We thus probed the limits of primitive αHA polymerization and polyester MMD assembly in terms of lowering initial reactant concentration and reaction volume as well as increased salinity. We selected a well-characterized αHA, PA, a structural analog of phenylalanine identified in various previous prebiotic studies,^58,59^ which has shown robust droplet assembly propensity in our previous works.^47−49,52,56,57^ As the previous studies involving primitive PA polymerization were generally performed in reactions at concentrations possibly inaccessible on early Earth (i.e., generally at 150–1000 mM and up to 500 μL^47−49,52,56,57^), we specified lower experimental parameters to test the limits of PA polymerization and MMD assembly down to 1 mM and 5 μL, as initial reactant concentration and reaction volume, respectively. Moreover, one of our previous studies^49^ showed that PA polymerization could occur in some salt chloride solutions (NaCl, KCl, and MgCl_2_) up to 100 mM, followed by clear MMD formation. We thus increased the salinity and tested PA polymerization in the presence of 1 M NaCl, KCl, and MgCl_2_. By probing the limits of which conditions could still facilitate the polymerization of αHAs and MMD assembly, we aimed to demonstrate that these chemical processes and reactions can occur even under lower concentrations and harsh conditions in the presence of salts, shedding more light on the plausibility of polyester synthesis and MMD assembly in the harsh and variable environments of early Earth, allowing us to gain a more realistic estimate of how widespread polyesters and polyester MMDs were on early Earth.

## Materials and Methods

### Chemicals

3-Phenyllactic acid (PA) (indeterminate chirality; *i.e*., a mixture of d- and l- forms) was purchased from Sigma-Aldrich (St. Louis, MO, USA). Acetonitrile (ACN) was purchased from Merck (Darmstadt, Germany) or Tokyo Chemical Industry (Chuo-ku, Tokyo, Japan). Sodium (NaCl), potassium (KCl), and magnesium (hexahydrate) (MgCl_2_) chlorides were purchased from Nacalai Tesque (Kyoto City, Kyoto, Japan). Ultrapure water for polyester syntheses and microdroplet generation was produced from a Barnstead Smart2Pure water filtration system (Thermo Fisher Scientific, Waltham, MA, USA).

### Synthesis

500 mM stock solutions of PA at 500 mM in ultrapure water were prepared, from which further dilutions in ultrapure water were performed to yield stock solutions used in various experiments (500, 50, 5, or 1 mM). The indicated volume of PA stock solution (500, 100, 20, or 5 μL) of the indicated concentration without pH modification was heated inside a 1.5 mL Eppendorf tube (Hamburg, Germany) with the cap open for 5–6 days (until complete dryness) at 80 °C in a Sahara 310 or Sahara 320 dry heating bath (Rocker Scientific, New Taipei City, Taiwan) in a draft chamber to mimic a primitive dehydrating environment. In particular, all experiments varying reactant concentration used a constant reactant volume of 500 μL. All experiments varying reactant volume used a constant reaction concentration of 500 mM. For experiments with salt, 250 μL of the respective 2 M stock solution was mixed with 250 μL of 500 mM PA, followed by the same dehydration synthesis method without pH modification.

### MALDI Sample Preparation and Analysis

MALDI-Time of Flight (ToF)-MS analysis was performed using an ultrafleXtreme Bruker Daltonics (Billerica, MA, USA) MALDI-ToF-MS in positive ion mode. External mass calibration was conducted using standard peptide mixtures provided by the user facility.

All samples without salt were dissolved in tetrahydrofuran (THF) to a concentration of 10 mg mL^–1^ (if the sample mass was known) or in 100 μL of total THF solvent (if the sample mass was unknown). For samples with salt, the samples were first dissolved in 9:1 THF:water to a concentration of 10 mg mL^–1^. Each sample was analyzed by using both DCTB (3-(4-*tert*-Butylphenyl)-2-methyl-2-propenylidene malononitrile) and CHCA (alpha-cyano-4-hydroxy-cinnamic acid) as sample matrices, which were also dissolved in THF to a concentration of 10 mg mL^–1^. The two matrices were utilized to cross-confirm and maximize spectral quality for all PA polymerization product samples under different conditions due to potential differences in ionization efficiency. Dissolved samples were mixed with the matrices (1:10 (v/v)) and applied to the plate for analysis. Each sample spectra were acquired in each matrix in singlicate (i.e., each sample was analyzed by MALDI-ToF-MS in duplicate in aggregate).

Monoisotopic peaks were then identified by isolating the highest intensity peak in an isotope envelope corresponding to a polymer product using a peak list generated from the mMass software (Open Source Software, Prague, Czech Republic) to subtract the matrix background signals (shown in Figure S1 for DCTB and Figure S2 for CHCA, Supporting Information). Major peaks were isolated by applying the following parameters: S/N > 10, absolute peak intensity >1000, and baselining (100 precision and 0 relative offset). Mass accuracy in ppm was calculated by comparing the observed mass with the calculated mass for major peaks corresponding to polymerization products. The intensities of all spectra peaks were plotted and normalized as follows. For the concentration and volume experiments, we normalized each spectrum based on the intensity of the highest peak observed across all conditions in the same set of experiments using the same matrix. For example, in the set of variable concentration experiments using the DCTB matrix, the highest intensity peak was observed in the 50 mM of PA reaction, and thus spectra starting from 5 mM and 1 mM of PA were normalized based on this peak intensity. In experiments with varying salts, the peaks were normalized based on the highest peak observed from each spectrum separately, whether the peak was identified as a product polymer peak or not. Detailed peak lists are presented in the Supporting Information (Tables S2–S17, Supporting Information); generally, only sodiated adducts (which are the most abundant and effectively demonstrate stepwise residue addition on a growing polyester polymer chain) are reported unless otherwise noted; for example, we use potassiated adducts for samples containing KCl.

### Center of Mass Estimation

The MALDI-ToF-MS spectra acquired from samples prepared at varying reactant concentrations (50 mM, 5 mM, or 1 mM) and reaction volume (100 μL, 20 μL, or 5 μL) were analyzed semiquantitatively, and the “center of mass” of each spectrum was estimated using a previously published method.^56^ The center of mass of a spectrum represents the polymer length/mass at which the summed polymer peak intensities of the sodiated adducts (M+Na^+^) below the center of mass equal the summed polymer peak intensities of the sodiated adducts above the center of mass. Note that this value does not take into account unpolymerized species (i.e., unreacted monomers). Specifically, the intensities of all detected sodiated adducts for a given spectrum were first summed (*I*_total_). For a given peak representing a polymer chain of n, the intensities of all sodiated adducts at lower or equal mass to itself were then also summed (*I*_*n*_). *I_n_*/*I*_total_ then represents the fraction of mass intensity (of the entire spectrum) below and including the given peak (*F*_*n*_). The peak at which *F*_*n*_ first exceeds 0.5 is defined as the approximate “center of mass”. However, because each peak may only increase up by one monomer unit at a time, this approximation may be too coarse, and a more accurate center of mass is calculated by subtracting a fractional offset value (*F*_*n*_ – 0.5)/(*F*_*n*_ – *F*_*n*–1_) from *n*; we also subtracted the fractional offset value 148.0524 × (*F*_*n*_ – 0.5)/(*F*_*n*_ – *F*_*n*–1_) from the *m*/*z* value of *n*, where 148.0524 represents the mass difference between adjacent polymer peaks. Because only sodiated peaks were analyzed in this manner and peak intensities of MALDI-ToF-MS are not quantitative in the absence of intensity standards, the actual center of mass may be different than estimated. However, this approximation allows a semiquantitative comparison of the degree of polymerization between samples.

### Microscopy

4:1 (v/v) water:ACN was added to each original reaction vessel after synthesis without pH adjustment, followed by vortexing and sonication; the rehydration volume was modulated depending on the original concentration and/or volume of the monomer reactants. For example, products of a 500 mM, 500 μL reaction were rehydrated with 500 μL water:ACN. Products of 500 mM reactions with variable reaction volumes (i.e., 100 μL, 20 μL, and 5 μL) and 500 μL reactions with variable reactant concentrations (i.e., 50 mM, 5 mM, or 1 mM) were rehydrated with 50–100 μL water:ACN. The Kuruma method of sample preparation was applied, which applies a double-sided tape (Naisutakku, Nichiban KK, Shinjuku-ku, Tokyo, Japan) containing a hole punched out to a 76 mm × 26 mm × 1 mm slide glass (Matsunami Glass, Kishiwada-shi, Osaka, Japan); the tape was stored in the refrigerator before use to preserve its adhesiveness. After the addition of 3–3.5 μL of each rehydrated sample to the vacated hole on the glass slide, a glass coverslip (No. 1 18 × 18 mm or similar, Matsunami Glass) was applied to cover the sample. Microscopy was performed on a DM5500 B upright epifluorescence microscope (HC PL FLUOTAR 40×/0.80 PH2 air objective, Leica, Wetzlar, Germany) with the Leica LAS X software and analyzed using FIJI (Fiji is Just ImageJ, http://fiji.sc). Observations were performed in duplicate or greater. Microdroplets observed in samples from all experimental conditions were compared with reference microscopy images of control samples. These control samples were prepared using PA polymerization reactions with a 500 mM reactant concentration and a 500 μL reaction volume, based on a previous study^48^ (shown in Figure S3, Supporting Information). Additionally, methods to determine the size distribution (using the software mentioned above) are detailed in Supplementary Method based on a previous work.^47−49,52,56,57^

## Results and Discussion

### The Effects of Lower Reactant Concentrations for PA Polymerization and Polyester Microdroplet Assembly

Our previous proof of principle experiments, with synthesis reactions generally conducted at 150–1000 mM and up to 500 μL monomer concentration/volume, showed clear polymerization and MMD formation,^47−49,52,56,57^ generally demonstrating the plausibility of αHA polymerization and MMD assembly through prebiotically plausible processes. However, these conditions, tested in laboratory settings, may have been unrealistic on prebiotic Earth, where such high initial concentrations of starting αHA reactants in the hundreds of millimoles range may not have been likely to exist in different primitive aqueous environments. For example, αHAs (i.e., LA) were detected in marine aerosol at ∼5.55 × 10^–10^ mM,^60^ while amino acids were detected in the ocean at ∼10^–4^ mM;^61^ these examples may indicate the dilution of analytes in primitive oceans following their synthesis. The diluted αHAs (low reactant concentration availability) and the large volume of ocean water (high volume of the reaction aqueous medium) would not have been suitable for polymerization. However, it may have been possible to achieve slightly higher concentrations of αHAs in the mM range outside of the early ocean. For example, both amino and hydroxy acids can be synthesized through electric discharge (e.g., the Miller–Urey experiment),^5,62^ and these products could have subsequently mixed into and accumulated to a significant degree either in localized areas in the ocean or in other smaller water bodies as well over time. It is also plausible that αHAs could also have accumulated via meteoritic delivery. For example, early Earth is estimated to have received approximately 10^15^ tons of meteorites,^63^ including carbonaceous chondrites containing varying levels of αHAs, during the Late Heavy Bombardment (∼3.9 billion years ago), which lasted for ∼100 million years.^39,64−66^ Both of these processes (electric discharge and meteoritic impacts) could have contributed significantly to the organic inventory on early Earth, including the availability of αHA monomers, thus potentially increasing αHA concentrations in various primitive aqueous environments, especially in smaller-scale water bodies such as lakes, ponds, tidal pools, puddles, hot springs, or even small pores in rocks^67^ which could have provided localized environments conducive to polymerization. These ancient geological bodies of water could have formed through a combination of meteorite impacts (possibly simultaneously delivering αHAs^10^ and creating craters^68^) and volcanic activity (e.g., calderas^69^). In particular, the ebb and flow of water at the shores of these smaller bodies of water or even larger oceans could also create small, bowl-like locales conducive to dehydration via evaporation through solar heating during day cycles^11^ or mild terrestrial surface temperatures caused by geothermal activity,^11,12^ leading to subsequent αHA polymerization. Once polymerized, polyesters can only assemble into MMDs upon rehydration,^48^ likely through wet–dry cycles,^11,12^ through the ebb and flow of water (i.e., tides and waves) or precipitation.^70,71^

Considering these varied ancient aqueous environments and the factors affecting polymerization and rehydration, it is thus essential to understand the specific conditions that favor both PA polymerization and polyester MMD assembly. The limited total mass of αHAs in smaller water bodies, even if high in αHA concentration, could result in much lower amounts of gross product polyester accumulation, which could be a hindrance to polyester MMD formation, a process that could require a large amount of material. A larger body of water with a more dilute αHA concentration could still accumulate more polyester products than a smaller, more concentrated (in αHAs) body of water, but dehydration in smaller volumes could convert the polyester products into a gel-like form before MMD assembly. Thus, it is crucial to investigate further the limits of the initial αHA reactant concentration and reaction volume that could initiate or be suitable for both PA polymerization and polyester MMD assembly.

**Figure 1:**
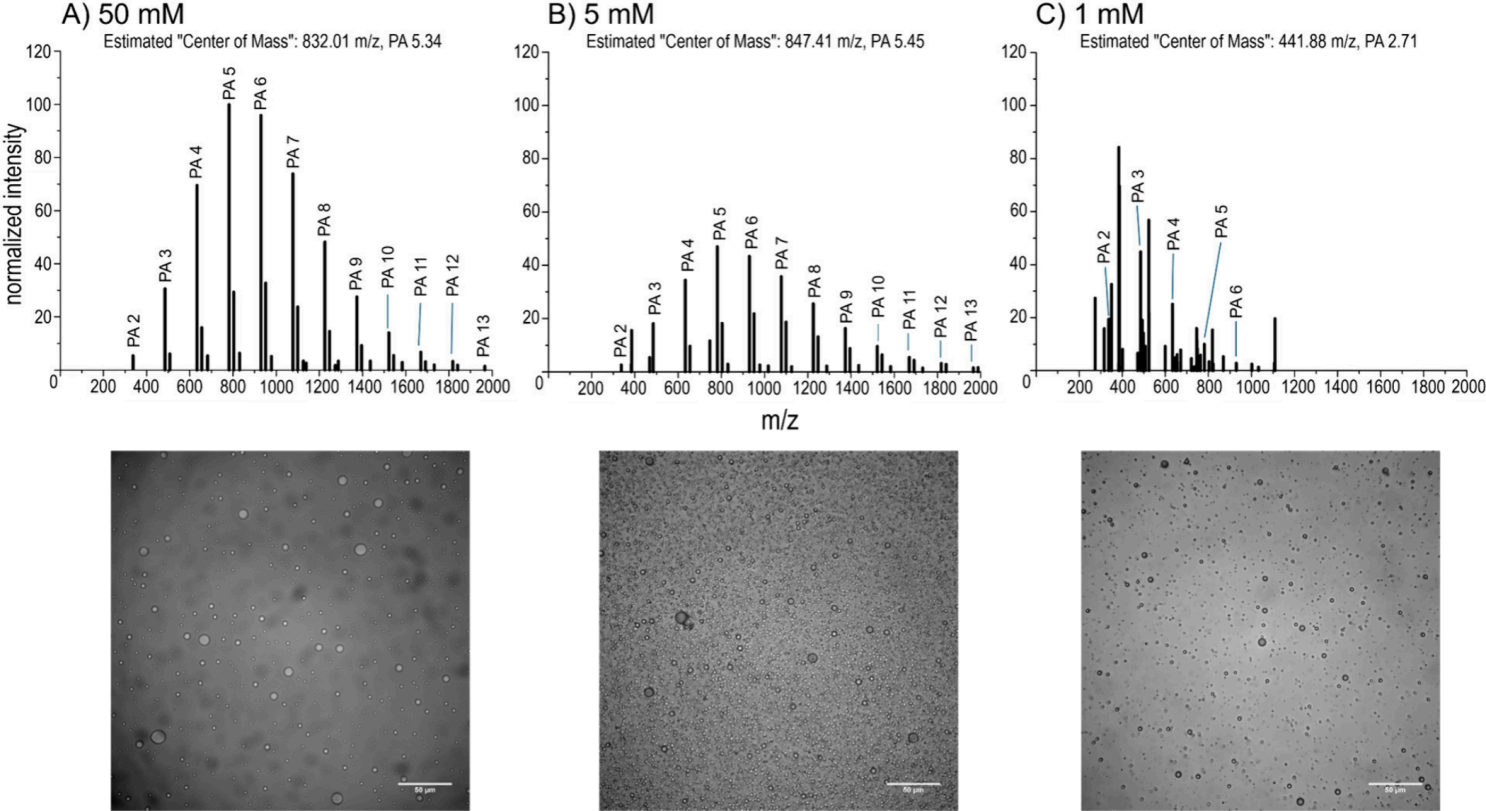
Representative MALDI-ToF-MS spectra using the DCTB matrix, the estimated center of mass (calculated from *F*_*n*_ of each product peak) of each spectrum, and the corresponding microscopy images of products from reactions containing variable initial PA concentrations of (A) 50 mM, (B) 5, and (C) 1 mM with a constant 500 μL reaction volume. Reaction products were confirmed using the CHCA matrix (Figure S4). Labeled peaks are sodiated (M+Na^+^); we exclusively analyzed sodiated peaks due to their abundance. See Figure S5 for more microscopy images. Scale bars: 50 μm.

In this study, we first lowered the initial PA concentration used in dehydration synthesis studies to confirm whether PA polymerization can still occur at lower initial reactant concentrations. We subjected PA to dehydration synthesis at 80 °C over several days (until dryness) to induce polymerization at initial reactant concentrations of 50 mM, 5 mM, and 1 mM at a constant reaction volume (500 μL). MALDI-ToF-MS analysis (using DCTB as the matrix) revealed that for initial reactant concentrations of 50 mM, 5 mM, and 1 mM, polymers up to 13-mers, 13-mers, and 6-mers, respectively, were observed (Figure 1, Tables S2–S4, Supporting Information). Evidence of PA polymerization is shown by an Δ148.05 Da mass ladder between adjacent peaks, which is attributed to water loss upon polymerization. Polymerization of the PA samples under the same condition was further confirmed by using a different matrix (CHCA, shown in Figure S4 and Tables S5–S7, Supporting Information). Lower initial PA reactant concentrations also appeared to qualitatively lead to a decrease in the average length of the resulting polymer products (Figure 1). Based on an estimation of the product center of mass calculated from normalized intensities of product peaks (*F*_*n*_), the lengths of the products produced from 1 mM PA reactions appear to be lower than those produced from 5 mM and 50 mM PA reactions. Nevertheless, it is still evident that PA polymerization can occur at a concentration as low as 1 mM.

Previous PA polymerization studies at higher initial reactant concentrations (500 mM) resulted in robust polyester MMD formation upon rehydration (Figure S3). In order to determine whether the polyester products yielded from reactions starting from lower reactant concentrations could also result in MMD assembly, we thus next performed microscopy analyses on the polyPA products formed via different reactant concentrations (50, 5, and 1 mM) after rehydration in an aqueous medium. The microscopy images clearly showed droplets in all conditions tested, including in products of reactions with initial PA concentrations as low as 1 mM, which matches well with the MALDI-ToF-MS data showing polyPA formation at this reactant concentration (Figure 1, more images in Figure S5). The observed MMD assembly suggests that polymerization of PA at concentrations as low as 1 mM is not a barrier for droplet assembly. Most MMDs had diameters generally within the single digit μm range, although no clear correlation MMD diameter with respect to differing reaction concentration was noticeable (shown in Figure S6, Supporting Information).

While previous experiments on the polymerization of αHAs via dehydration^47−49,52,56,57^ used high initial reactant concentrations to ensure robust detection of polymers via MALDI-ToF-MS, our observations show that even at PA concentrations as low as 1 mM, polymerization and MMD assembly can still occur. It is worth noting that intensity calibration using internal standard was not performed in this study to compare the ion intensities between samples. However, it was assumed that the target of the analysis, polyPA, exhibits largely consistent ionization properties under the same analytical conditions. Overall, this finding opens up the possibility of future investigations into even lower reactant concentrations (e.g., into the μM range) to determine the minimum levels at which PA polymerization and subsequent polyester MMD formation can still occur.

### The Effects of Lower Reaction Volumes for PA Polymerization and Polyester Microdroplet Formation

Smaller water bodies such as puddles, rock pores, or even pools at the banks of lakes or oceans are conducive to αHA polymerization via natural wet–dry cycles. This localized evaporation process could result in the reaction volume decreasing significantly while simultaneously increasing the reactant concentration. Similarly, rock pores could also potentially accumulate high concentrations of αHA reactants in small volumes, such as, as a result of thermophoretic activity.^72^ Thus, we next assessed PA polymerization against decreasing initial reaction volumes. In particular, as PA polymerization and MMD assembly at 500 μL (with PA concentrations as low as 1 mM) were successfully observed, we surmised that lowering the reaction volume down to as low as 5 μL (which can be reached within mm-range rock pores),^72^ with a constant 500 mM PA concentration could still result in enough PA starting materials to enable robust PA polymerization and MMD assembly.

**Figure 2:**
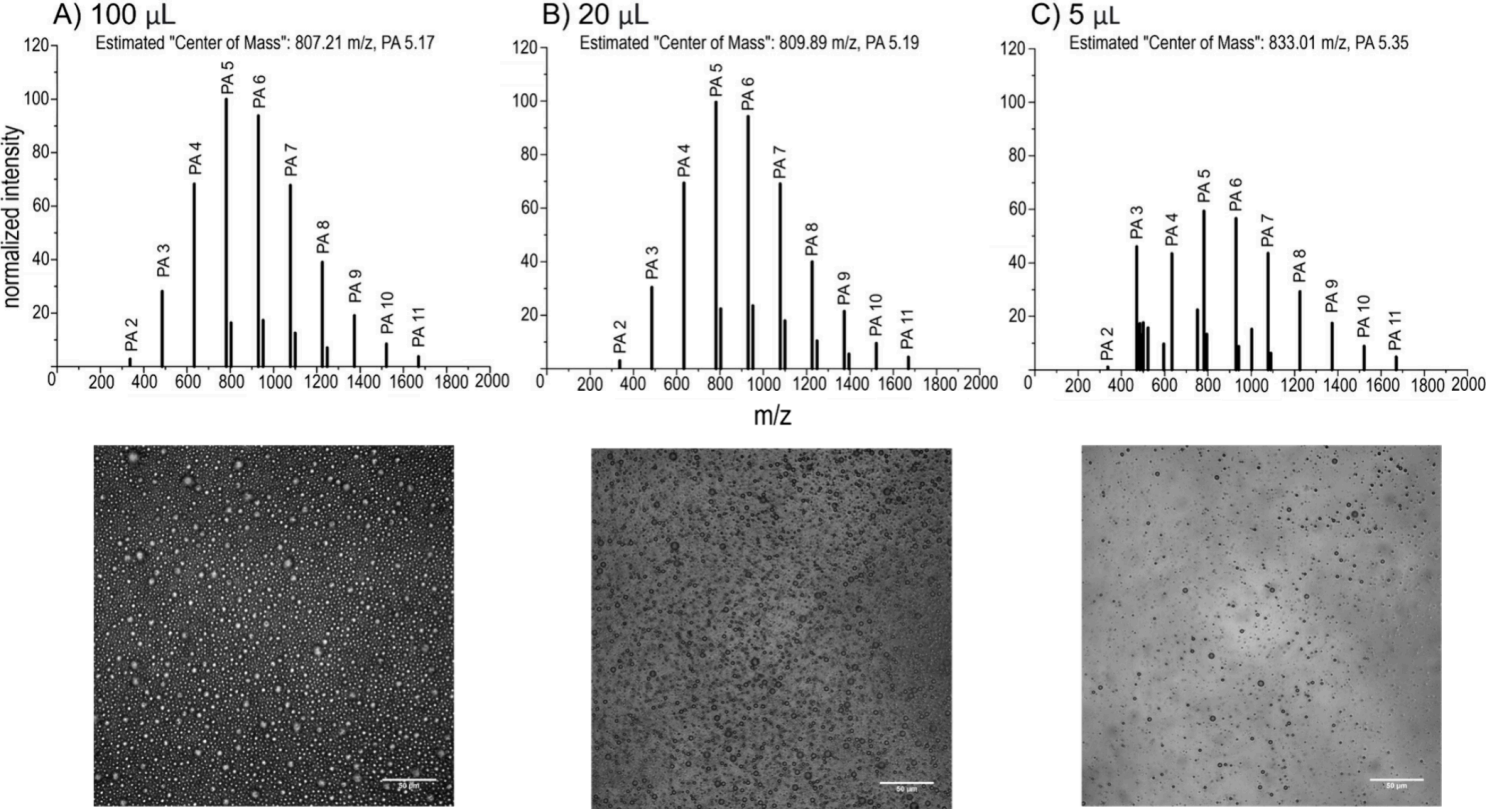
Representative MALDI-ToF-MS spectra using the DCTB matrix, the estimated center of mass (calculated from *F*_*n*_ of each product peak) of each spectrum, and the corresponding microscopy images of products from variable initial volume ((A) 100 μL, (B) 20 μL, and (C) 5 μL) PA polymerization reactions (at a constant 500 mM reactant concentration). Reaction products were confirmed using the CHCA matrix (Figure S7). Labeled peaks are sodiated (M+Na^+^); we exclusively analyzed sodiated peaks due to their abundance. See Figure S8 for more microscopy images. Scale bars: 50 μm.

Similar to the reactions varying initial reactant concentration, we first subjected PA to dehydration synthesis at 80 °C over several days to induce polymerization at various initial reaction volumes (100 μL, 20 μL, and 5 μL) at a constant PA concentration (500 mM). MALDI-ToF-MS (DCTB matrix) analysis resulted in the detection of PA polymer products ranging up to 11-mers across all tested sample volumes (Figure 2, Tables S8–S10, Supporting Information). This MS observation was further confirmed using the CHCA matrix (shown in Figure S7 and Tables S11–S13, Supporting Information). These findings indicate that the polymerization of PA can occur effectively even at initial reaction volumes as low as 5 μL without showing any significant difference in average polymer length compared to the 100 and 20 μL reactions (based on the estimated center of mass calculation using *F*_*n*_ of product peaks) (Figure 2). A low volume of water, such as 5 μL, can be fully evaporated rapidly, especially compared to 100 or 20 μL reactions, suggesting that larger reactant volumes may allow the polymerization reaction to yield more or longer polymers due to reaction time. However, even incomplete evaporation would increase analyte concentration, potentially resulting in further concentrating of αHAs and allowing polymerization to occur in fully dry conditions once the reaction solution has been fully converted to the product gel phase; a study by Yi et al.^73^ confirms the plausibility of this dry-phase process for similar dehydration synthesis processes of glycol nucleic acid (GNA) monomers and various dicarboxylic acids (DCAs). Finally, the apparent lack of difference in average product polymer length regardless of reaction volume, as demonstrated by the estimation of the center of mass of each product spectrum, is in contrast with the variable reactant concentration studies, which showed shorter product polymers at lower reactant concentrations. This finding suggests that perhaps reactant concentration is more of a predictor of product length than of reaction volume.

Next, we performed microscopy analyses and the images also clearly showed droplet assembly in all conditions (Figure 2, Figure S8), including in reaction volumes as low as 5 μL, which appears to correspond with the MS data indicating that robust PA polymerization occurred in all conditions. Similar to the concentration parameter mentioned above, no clear correlation was observed between MMD diameters with respect to differing reaction volumes (shown in Figure S6, Supporting Information).

Overall, these findings suggest that even water bodies on ancient Earth holding miniscule volumes may not be a barrier to PA polymerization and subsequent MMD assembly, as long as αHAs can accumulate to a sufficient concentration. For example, mm-scale rock pores, such as those that have been shown to be prebiotically relevant through their ability to concentrate nucleic acids and drive coacervate evolution, would be in a similar volume regime as those probed in this study.^72,74^ αHA polymerization and subsequent polyester MMD assembly within such rock pores, which can house submicroliter volumes of aqueous solvent (down to the subnanoliter range),^75^ could be targets of future research that would put the results from the current study into a clearer geological context at the origins of life.

### The Effects of Higher Solution Salinity for PA Polymerization and Polyester Microdroplet Formation

Various salts and salt-derived ions are required for biological functionality. For example, Na^+^ is crucial in the regulation of protein synthesis within the cell nucleus,^76^ K^+^ is important in cells’ replication,^77^ and Mg^2+^ is essential for RNA folding and functions;^78^ each cation contributes many more functions than the ones listed here. As a result, the balance of cations within the cell is crucial for balancing intracellular processes, partially overseen by the sodium–potassium pump.^79^ Additionally, the typical concentration of these cations in a cell will differ based on their types and functionalities, although intracellular concentrations of K^+^, Na^+^, and Mg^2+^ are generally in the quite wide range of 0.003–0.31 M;^80^ for example, the concentrations of Na^+^, K^+^, and Mg^2+^ in human HeLa cells are 0.066, 0.064, and 0.015 M, respectively.^80^

Given that salts are essential to modern biochemical processes, it is also true that the same salts would have been essential to primitive cells, i.e., protocells, and other prebiotic processes. For example, Na^+^ affects vesicle membrane rigidity,^81^ low concentrations of K^+^ can preferentially be uptaken in polyester protocells,^49^ and Mg^2+^ is required for ribozyme function.^82^ Thus, the abundance and availability of such salts on early Earth would have driven the direction of primitive evolution. The concentrations of oceanic Na^+^, K^+^, and Mg^2+^ (generally as chloride salts) have varied significantly over geological time scales due to various factors, including tectonic activity^83^ and hydrothermal processes.^84^ In modern oceans, total salinity is roughly 35 g/L;^85^ Na^+^, K^+^, and Mg^2+^ cations are thus observed in estimated concentration regimes in the range of 0.01–0.46 M, with Na^**+**^ being the most abundant among the trio of cations, and K^+^ being the least abundant.^86,87^ However, exact salinities within ancient oceans have been debated, with various estimations that the ancient ocean would have been saltier in general than those of modern oceans by up to 2-fold. For instance, in the ancient ocean, the concentration of Na^+^ is estimated to be up to 1.1 M.^88^ However, the concentration of ancient oceanic K^+^ is generally believed to have been lower than that of modern oceans, possibly due to their existence within various minerals. Models of ancient oceanic Mg^2+^ suggest a concentration between 10–50 mM, depending on the time range.^89^ Nevertheless, there would still be some variability depending on the body of water, which could result in much higher salinities, such as in the Dead Sea^90^ or in hypersaline oceanic brines^91^ on modern Earth; we can expect similar variability on ancient Earth. In particular, ancient evaporative processes at or near these bodies of water could have resulted in localized environments with extreme salinities upward of the molar range, while primitive K^+^ could have accumulated to high degrees within mica sheets.^92^ Therefore, probing PA polymerization and subsequent polyester MMD assembly under high salt conditions could reveal how plausible these processes were under various conditions on early Earth.

**Figure 3:**
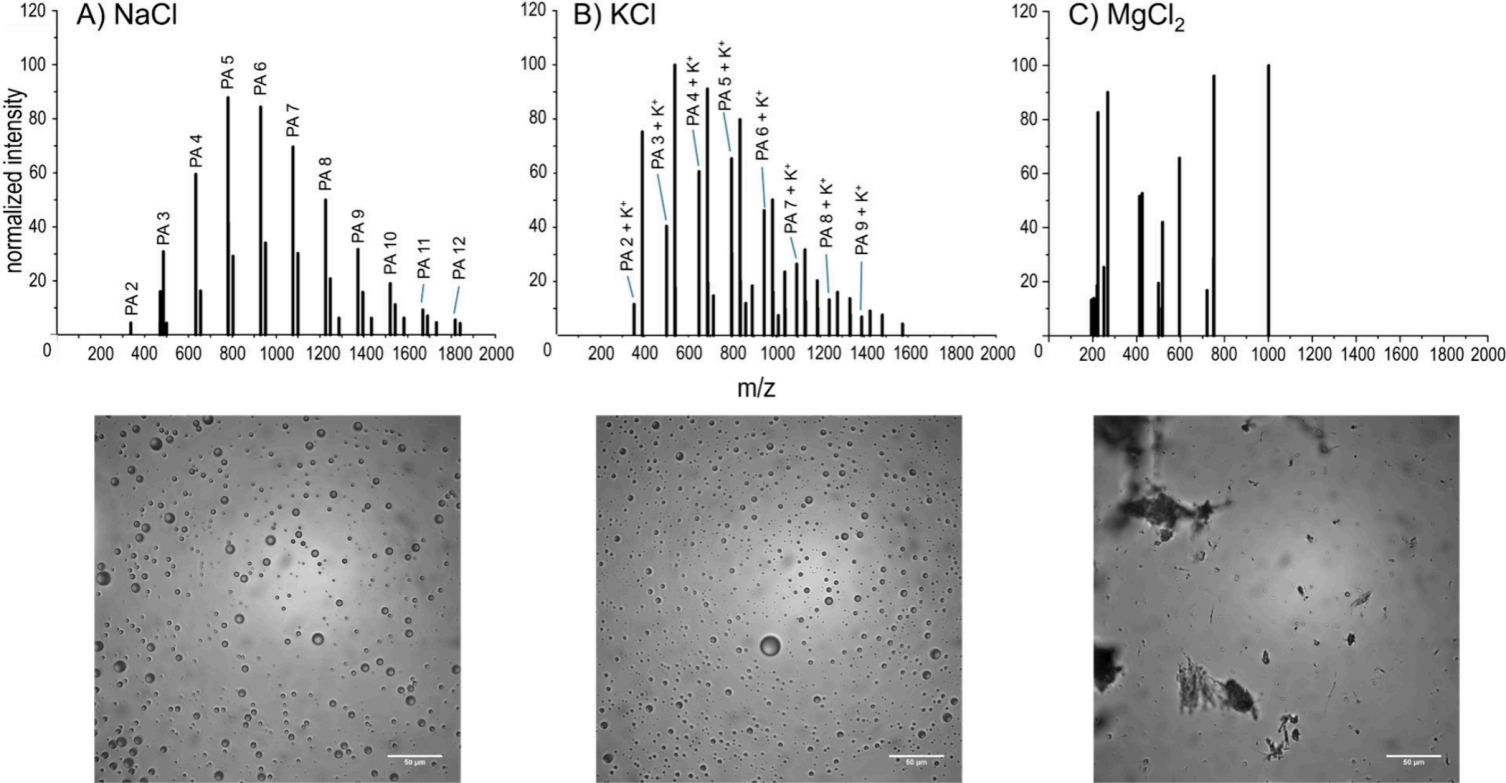
Representative MALDI-ToF-MS spectra (using the DCTB matrix) and the corresponding microscopy images of products from 500 mM PA polymerization reactions (at constant 500 μL reaction volume) in the presence of 1 M (A) NaCl, (B) KCl, and (C) MgCl_2_. Reaction products were confirmed using the CHCA matrix (Figure S9). Labeled peaks are sodiated (M+Na^+^) (A) or potassiated (M+K^+^) (B). No polymerization products were detected in (C). The peaks were normalized based on the intensity of the highest peak (not necessarily a polymer product peak) observed from each spectra separately. Scale bars: 50 μm.

Our previous work^49^ demonstrated successful PA polymerization and subsequent MMD assembly in salt solutions of up to 100 mM of NaCl, KCl, and MgCl_2_. However, the study also showed reduced PA polymerization in a 100 mM CaCl_2_ solution, with no MMD formation, suggesting that polymerization and MMD assembly were limited to lower CaCl_2_ environments (a 10 mM CaCl_2_ solution allowed PA polymerization and MMD assembly). This finding indicates that PA polymerization can be inhibited at higher salt concentrations. Even though PA polymerization and subsequent MMD assembly could occur at 100 mM NaCl, KCl, and MgCl_2_, it is possible that higher concentrations of each could result in inhibition of these processes, similar to what was observed when CaCl_2_ concentration increased up to 100 mM. Therefore, to test the limits of PA polymerization and subsequent MMD assembly against high salt concentrations in the reaction medium, we subjected PA monomers (500 μL, 500 mM) to dehydration synthesis at 80 °C over several days in the presence of 1 M NaCl, KCl, or MgCl_2_. MALDI-ToF-MS (DCTB matrix) (Figure 3, Tables S14–S15, Supporting Information) data revealed that 1 M NaCl and KCl did not inhibit polymerization, with product polymers of up to 12-mers and 9-mers, respectively, observed. However, 1 M MgCl_2_ appeared to significantly inhibit polymerization, with no observed polymerization products; these observations were confirmed by MALDI-ToF-MS using a CHCA matrix (Figure S9 and Tables S16–S17, Supporting Information). We speculate that when MgCl_2_ is dissolved in water, free Mg^2+^ ions could possibly strongly coordinate with the carboxyl and hydroxyl groups of the PA monomers, stabilizing the monomers and reducing their reactivity,^93^ thus preventing the formation of ester bonds necessary for polymerization; in contrast, the monovalent Na^+^ and K^+^ may not be able to coordinate as strongly as Mg^2+^. A similar phenomenon may have been observed in CaCl_2_-containing reactions (with Ca^2+^ being possibly a stronger PA binder than Mg^2+^), which were previously shown to inhibit PA polymerization even at 100 mM.^49^ With the caveat that desalting was not performed in these analyses, suppression in MALDI-ToF-MS due to high salt content may have reduced the ion intensities of the polyPA products. However, the formation of MMDs (discussed in the next paragraph) in NaCl and KCl samples, and the absence of MMDs in the MgCl_2_ sample corroborated the presence of polyPA in the respective MALDI-ToF-MS spectra for NaCl- and KCl-containing reactions and its absence in the MALDI-ToF-MS spectrum for MgCl_2_-containing reactions. Nevertheless, this limitation creates an opportunity for further evaluation in future studies using high-performance liquid chromatography (HPLC) or size exclusion chromatography (SEC).

Microscopy images to detect whether MMDs could assemble following rehydration were then performed to reveal the feasibility of PA polymers synthesized in the presence of 1 M salt solutions (NaCl, KCl, and MgCl_2_). PA polymerization products in the presence of 1 M NaCl and 1 M KCl resulted in robust MMD assembly (Figure 3A,B), which matched the MALDI-ToF-MS data, indicating the presence of a number of polyPA products synthesized under these saline conditions. However, the PA polymerization reaction in the presence of 1 M MgCl_2_ did not appear to result in robust MMD assembly (Figure 3C), matching MALDI-ToF-MS results which did not show accumulation of polymerization products; instead, aggregates were observed (more images in Figure S10, Supporting Information). However, some small particle-like structures were also occasionally observed, which could not be confirmed to be spherical droplets when compared with the MMDs from the control sample (Figure S3, Supporting Information). However, we cannot completely rule out the possibility of some minor basal levels of polyPA polymer production in the presence of MgCl_2_ (despite no detected polymers being detected via MALDI-ToF-MS). The fact that we did not observe robust spherical droplet assembly in the presence of 1 M MgCl_2,_ especially compared to all other conditions probed in this study indicates that very few polymers, if any, were produced. Similarly to the parameters tested above, no significant correlation was observed between MMD diameter and salt identity (shown in Figure S6, Supporting Information).

Hence, our findings indicate that the type of salt ion significantly influences polyester and MMD availability on early Earth, with the PA-based system tolerant to certain ionic environments (i.e., high NaCl or KCl) and inhibited in others (i.e., high in MgCl_2_). For example, Europa, one of Jupiter’s water-containing moons, contains NaCl (as well as some KCl) on the surface (which also shows evidence of water),^94,95^ possibly resulting in an environment reflecting ionic conditions tolerable for αHA polymerization and subsequent MMD assembly; however, some calcium may also exist on Europa’s surface, which could be inhibitory to these processes depending on concentration. In contrast, modeling and environmental simulation studies predict coexistence of NaCl and MgCl_2_ (although they have yet to be unequivocally detected *in situ*) in aqueous brines the Martian surface,^96^ which could be inhibitory for αHA polymerization and subsequent MMD assembly in elevated MgCl_2_ conditions. Extending this to exoplanets, the HD209458b exoplanet, which has shown some evidence of the existence of water,^97^ contains MgCl_2_ in its atmosphere, which would also be inhibitory to αHA polymerization and MMD assembly if the ions also existed in the solution phase on the planetary surface. Meanwhile, on Earth today, geological lakes have a wide range of salinity profiles, which could also be expected on early Earth. For example, lakes rich in potash (minerals which contain potassium), such as those in Australia, Argentina, Bolivia, China, *etc.,*(98) may offer environments tolerable for αHA polymerization and MMD assembly. In contrast, locations with high magnesium content, such as Lake Salda in Turkey,^99^ are likely to inhibit αHA polymerization.

## Conclusion

Investigating the limits of PA polymerization and subsequent polyester microdroplet assembly under varied prebiotic conditions here, including low initial reactant concentrations, low initial reaction volumes, and high salt concentrations, suggests the feasibility of prebiotic polyester synthesis and MMD assembly in a wider range of conditions than previously known. In particular, PA reaction volumes as low as 5 μL and reactant concentrations as low as 1 mM still resulted in robust polymerization and droplet assembly, expanding previous studies that used much higher initial reactant concentrations and volumes more reflective of laboratory conditions rather than those that would have been available on primordial Earth. It is plausible that even lower reaction volumes or reactant concentrations than those tested here could also still be conducive to primitive polyester synthesis and MMD assembly, such as those found in oceanic rock pores or even aerosols, which could be the focus of future work. PA polymerization reactions probed in this study were all performed at 80 °C, and variation in the evaporation rate affected by varying reaction temperatures, especially in reactions of variable volume, could result in further differences in PA polymerization. Additionally, NaCl and KCl, even at 1 M, had no significant inhibitory effect on PA polymerization or subsequent droplet assembly, while 1 M MgCl_2_ appeared to inhibit polyester polymerization and MMD formation, which gives more clues into the salt-requirements of primitive environments that housed such reactions and processes. Collectively, expanding the current study to probe the polymerization and subsequent MMD assembly of other prebiotically relevant αHAs with a lower MMD assembly propensity than PA, such as aliphatic or charged αHAs (such as GA, LA, or malic acid), will provide a clearer picture into the whole prebiotic chemical inventory available for αHA-based processes relevant to the origins of life on early Earth and potential exoplanets.

## Supporting Information Available

Detailed descriptions of experimental methods, including droplet size image analysis using FIJI; Supplementary figures presenting MALDI-ToF-MS spectra, microscopy images, and histograms of droplet size distributions under varying experimental conditions; Supplementary tables summarizing experimental conditions, peaklists for PA polymer products sorted by mass, and reaction outcomes under different matrices and salt concentrations (PDF)
Raw data, including MALDI spectra, peaklists, and microscopy images categorized by variations in reactant concentration, volume, and salt presence (ZIP)

## References

[ref1] Zaritsky A. R.; Grachev V. I.; Vorontsov Y. P.; Pronin V. S. Abiogenesis Transition from the Atmosphere into the Hydrosphere: From Vesicles to Protocells. Radioelectron. Nanosyst. Inf. Technol. 2014, 221–231. 10.17725/RENSITe.0006.201412f.0221.

[ref2] Mann S. Systems of Creation: The Emergence of Life from Nonliving Matter. Acc. Chem. Res. 2012, 45 (12), 2131–2141. 10.1021/ar200281t. 22404166

[ref3] Kitadai N.; Maruyama S. Origins of Building Blocks of Life: A Review. Geosci. Front. 2018, 9 (4), 1117–1153. 10.1016/j.gsf.2017.07.007.

[ref4] Glansdorff N.; Xu Y.; Labedan B. The Last Universal Common Ancestor: Emergence, Constitution and Genetic Legacy of an Elusive Forerunner. Biol. Direct 2008, 3, 29 10.1186/1745-6150-3-29. 18613974 PMC2478661

[ref5] Miller S. L. Production of Some Organic Compounds under Possible Primitive Earth Conditions1. J. Am. Chem. Soc. 1955, 77 (9), 2351–2361. 10.1021/ja01614a001.

[ref6] Rimmer P. B.; Shorttle O. Origin of Life’s Building Blocks in Carbon- and Nitrogen-Rich Surface Hydrothermal Vents. Life 2019, 9 (1), 12 10.3390/life9010012. 30682803 PMC6463091

[ref7] Martin W.; Baross J.; Kelley D.; Russell M. J. Hydrothermal Vents and the Origin of Life. Nat. Rev. Microbiol. 2008, 6 (11), 805–814. 10.1038/nrmicro1991. 18820700

[ref8] Vincent L.; Colón-Santos S.; Cleaves J. H. II; Baum D. A.; Maurer S. E. The Prebiotic Kitchen: A Guide to Composing Prebiotic Soup Recipes to Test Origins of Life Hypotheses. Life 2021, 11 (11), 1221 10.3390/life11111221. 34833097 PMC8618940

[ref9] Pearce B. K. D.; Molaverdikhani K.; Pudritz R. E.; Henning T.; Cerrillo K. E. Toward RNA Life on Early Earth: From Atmospheric HCN to Biomolecule Production in Warm Little Ponds. Astrophys. J. 2022, 932 (1), 9 10.3847/1538-4357/ac47a1.

[ref10] Sithamparam M.; Satthiyasilan N.; Chen C.; Jia T. Z.; Chandru K. A Material-Based Panspermia Hypothesis: The Potential of Polymer Gels and Membraneless Droplets. Biopolymers 2022, 113 (5), e23486 10.1002/bip.23486. 35148427

[ref11] Hud N. V.; Anet F. A. Intercalation-Mediated Synthesis and Replication: A New Approach to the Origin of Life. J. Theor. Biol. 2000, 205 (4), 543–562. 10.1006/jtbi.2000.2084. 10931751

[ref12] Senatore F; Serra R.; Villani M. Modelling Wet-Dry Cycles in the Binary Polymer Model. In Communications in Computer and Information Science; Springer, 2023; Vol. 1780, pp 119–129.

[ref13] Subbotin V.; Fiksel G. Exploring the Lipid World Hypothesis: A Novel Scenario of Self-Sustained Darwinian Evolution of the Liposomes. Astrobiology 2023, 23 (3), 344–357. 10.1089/ast.2021.0161. 36716277 PMC9986030

[ref14] Muchowska K. B.; Varma S. J.; Moran J. Nonenzymatic Metabolic Reactions and Life’s Origins. Chem. Rev. 2020, 120 (15), 7708–7744. 10.1021/acs.chemrev.0c00191. 32687326

[ref15] Mojarro A.; Jin L.; Szostak J. W.; Head J. W. 3rd; Zuber M. T. In Search of the RNA World on Mars. Geobiology 2021, 19 (3), 307–321. 10.1111/gbi.12433. 33565260 PMC8248371

[ref16] Hess B. L.; Piazolo S.; Harvey J. Lightning Strikes as a Major Facilitator of Prebiotic Phosphorus Reduction on Early Earth. Nat. Commun. 2021, 12 (1), 1535 10.1038/s41467-021-21849-2. 33727565 PMC7966383

[ref17] Tharumen N.; Sithamparam M.; Satthiyasilan N.; Bahari S. A.; Chelvanathan P.; Latip J.; Mat Jusoh Hussain A. H.; Abdullah M.; Chandru K. The Role of the Faint Young Sun in Prebiotic Chemistry: A Chemical Perspective on Prebiotic Polymerization. Jurnal Kejuruteraan 2024, 36 (3), 1311–1321. 10.17576/jkukm-2024-36(3)-39.

[ref18] Ershov B. G. Natural Radioactivity of the Crust as an Important Factor of the Chemical Evolution of the Early Earth. Precambrian Res. 2024, 406, 107400 10.1016/j.precamres.2024.107400.

[ref19] Zagórski Z. P.; Kornacka E. M. Ionizing Radiation: Friend or Foe of the Origins of Life?. Orig. Life Evol. Biosph. 2012, 42 (5), 503–505. 10.1007/s11084-012-9314-1. 23080010 PMC3517802

[ref20] Kobayashi K.; Ise J.-I.; Aoki R.; Kinoshita M.; Naito K.; Udo T.; Kunwar B.; Takahashi J.-I.; Shibata H.; Mita H.; Fukuda H.; Oguri Y.; Kawamura K.; Kebukawa Y.; Airapetian V. S. Formation of Amino Acids and Carboxylic Acids in Weakly Reducing Planetary Atmospheres by Solar Energetic Particles from the Young Sun. Life 2023, 13 (5), 1103 10.3390/life13051103. 37240748 PMC10221653

[ref21] Gaylor M. O.; Miro P.; Vlaisavljevich B.; Kondage A. A. S.; Barge L. M.; Omran A.; Videau P.; Swenson V. A.; Leinen L. J.; Fitch N. W.; Cole K. L.; Stone C.; Drummond S. M.; Rageth K.; Dewitt L. R.; González Henao S.; Karanauskus V. Plausible Emergence and Self Assembly of a Primitive Phospholipid from Reduced Phosphorus on the Primordial Earth. Orig. Life Evol. Biosph. 2021, 51 (3), 185–213. 10.1007/s11084-021-09613-4. 34279769

[ref22] Hirakawa Y.; Kakegawa T.; Furukawa Y. Borate-Guided Ribose Phosphorylation for Prebiotic Nucleotide Synthesis. Sci. Rep. 2022, 12 (1), 11828 10.1038/s41598-022-15753-y. 35853897 PMC9296462

[ref23] Ritson D.; Sutherland J. D. Prebiotic Synthesis of Simple Sugars by Photoredox Systems Chemistry. Nat. Chem. 2012, 4 (11), 895–899. 10.1038/nchem.1467. 23089863 PMC3589744

[ref24] Gull M.; Feng T.; Cruz H. A.; Krishnamurthy R.; Pasek M. A. Prebiotic Chemistry of Phosphite: Mild Thermal Routes to Form Condensed-P Energy Currency Molecules Leading Up to the Formation of Organophosphorus Compounds. Life 2023, 13 (4), 920 10.3390/life13040920. 37109449 PMC10144983

[ref25] Chandru K.; Mamajanov I.; Cleaves J. H. II; Jia T. Z. Polyesters as a Model System for Building Primitive Biologies from Non-Biological Prebiotic Chemistry. Life 2020, 10 (1), 6 10.3390/life10010006. 31963928 PMC7175156

[ref26] Chandru K.; Potiszil C.; Jia T. Z. Alternative Pathways in Astrobiology: Reviewing and Synthesizing Contingency and Non-Biomolecular Origins of Terrestrial and Extraterrestrial Life. Life 2024, 14 (9), 1069 10.3390/life14091069. 39337854 PMC11433091

[ref27] Williams N. C.; O’Neill L. A. J. A Role for the Krebs Cycle Intermediate Citrate in Metabolic Reprogramming in Innate Immunity and Inflammation. Front. Immunol. 2018, 9, 141 10.3389/fimmu.2018.00141. 29459863 PMC5807345

[ref28] Omini J.; Wojciechowska I.; Skirycz A.; Moriyama H.; Obata T. Association of the Malate Dehydrogenase-Citrate Synthase Metabolon Is Modulated by Intermediates of the Krebs Tricarboxylic Acid Cycle. Sci. Rep. 2021, 11 (1), 18770 10.1038/s41598-021-98314-z. 34548590 PMC8455617

[ref29] Li X.; Yang Y.; Zhang B.; Lin X.; Fu X.; An Y.; Zou Y.; Wang J.-X.; Wang Z.; Yu T. Lactate Metabolism in Human Health and Disease. Signal Transduct. Target Ther. 2022, 7 (1), 305 10.1038/s41392-022-01151-3. 36050306 PMC9434547

[ref30] Ohta A.; Murakami H.; Higashimura E.; Suga H. Synthesis of Polyester by Means of Genetic Code Reprogramming. Chem. Biol. 2007, 14 (12), 1315–1322. 10.1016/j.chembiol.2007.10.015. 18096500

[ref31] Ibrahim S. A.; Ayivi R. D.; Zimmerman T.; Siddiqui S. A.; Altemimi A. B.; Fidan H.; Esatbeyoglu T.; Bakhshayesh R. V. Lactic Acid Bacteria as Antimicrobial Agents: Food Safety and Microbial Food Spoilage Prevention. Foods 2021, 10 (12), 3131 10.3390/foods10123131. 34945682 PMC8701396

[ref32] Park K. M.; Yoon S.-G.; Choi T.-H.; Kim H. J.; Park K. J.; Koo M. The Bactericidal Effect of a Combination of Food-Grade Compounds and Their Application as Alternative Antibacterial Agents for Food Contact Surfaces. Foods 2020, 9 (1), 59 10.3390/foods9010059. 31936035 PMC7022224

[ref33] Padhi S.; Sharma S.; Sahoo D.; Montet D.; Rai A. K. Potential of Lactic Acid Bacteria as Starter Cultures for Food Fermentation and as Producers of Biochemicals for Value Addition. In Lactic Acid Bacteria in Food Biotechnology; Ray R. C., Paramithiotis S., de Carvalho Azevedo V. A., Montet D., Eds.; Elsevier, 2022; pp 281–304.

[ref34] Fiume M. M. Alpha Hydroxy Acids. Int. J. Toxicol. 2017, 36 (5_suppl2), 15S–21S. 10.1177/1091581817716656. 29025346

[ref35] Valle-González E. R.; Jackman J. A.; Yoon B. K.; Mokrzecka N.; Cho N.-J. pH-Dependent Antibacterial Activity of Glycolic Acid: Implications for Anti-Acne Formulations. Sci. Rep. 2020, 10 (1), 7491 10.1038/s41598-020-64545-9. 32367064 PMC7198592

[ref36] Vlachopoulos A.; Karlioti G.; Balla E.; Daniilidis V.; Kalamas T.; Stefanidou M.; Bikiaris N. D.; Christodoulou E.; Koumentakou I.; Karavas E.; Bikiaris D. N. Poly(Lactic Acid)-Based Microparticles for Drug Delivery Applications: An Overview of Recent Advances. Pharmaceutics 2022, 14 (2), 359 10.3390/pharmaceutics14020359. 35214091 PMC8877458

[ref37] Irfan J.; Ali A.; Hussain M. A.; Haseeb M. T.; Naeem-Ul-Hassan M.; Hussain S. Z. Citric Acid Cross-Linking of a Hydrogel from Aloe Vera (Aloe Barbadensis M.) Engenders a pH-Responsive, Superporous, and Smart Material for Drug Delivery. RSC Adv. 2024, 14 (12), 8018–8027. 10.1039/D4RA00095A. 38454944 PMC10918532

[ref38] Sun X.-J.; Zhang X.; Dong H.; Yang D.-D.; Tang H.-L.; Zhai Y.-C.; Wei J.-Z.; Zhang F.-M. Porous Metal–organic Gel Assisted by L-Tartaric Acid Ligand for Efficient and Controllable Drug Delivery. New J. Chem. 2018, 42 (18), 14789–14795. 10.1039/C8NJ02007H.

[ref39] Pizzarello S.; Wang Y.; Chaban G. M. A Comparative Study of the Hydroxy Acids from the Murchison, GRA 95229 and LAP 02342 Meteorites. Geochim. Cosmochim. Acta 2010, 74 (21), 6206–6217. 10.1016/j.gca.2010.08.013.

[ref40] Zang X.; Ueno Y.; Kitadai N. Photochemical Synthesis of Ammonia and Amino Acids from Nitrous Oxide. Astrobiology 2022, 22 (4), 387–398. 10.1089/ast.2021.0064. 35196128

[ref41] Huber C.; Eisenreich W.; Wächtershäuser G. Synthesis of α-Amino and α-Hydroxy Acids under Volcanic Conditions: Implications for the Origin of Life. Tetrahedron Lett. 2010, 51 (7), 1069–1071. 10.1016/j.tetlet.2009.12.084.

[ref42] Kuisle O.; Quiñoá E.; Riguera R. Solid Phase Synthesis of Depsides and Depsipeptides. Tetrahedron Lett. 1999, 40 (6), 1203–1206. 10.1016/S0040-4039(98)02566-0. 11674717

[ref43] Forsythe J. G.; Petrov A. S.; Millar W. C.; Yu S.-S.; Krishnamurthy R.; Grover M. A.; Hud N. V.; Fernández F. M. Surveying the Sequence Diversity of Model Prebiotic Peptides by Mass Spectrometry. Proc. Natl. Acad. Sci. U. S. A. 2017, 114 (37), E7652–E7659. 10.1073/pnas.1711631114. 28847940 PMC5604043

[ref44] Fried S. D.; Fujishima K.; Makarov M.; Cherepashuk I.; Hlouchova K. Peptides before and during the Nucleotide World: An Origins Story Emphasizing Cooperation between Proteins and Nucleic Acids. J. R. Soc. Interface 2022, 19 (187), 20210641 10.1098/rsif.2021.0641. 35135297 PMC8833103

[ref45] Fialho D. M.; Karunakaran S. C.; Greeson K. W.; Martínez I.; Schuster G. B.; Krishnamurthy R.; Hud N. V. Depsipeptide Nucleic Acids: Prebiotic Formation, Oligomerization, and Self-Assembly of a New Proto-Nucleic Acid Candidate. J. Am. Chem. Soc. 2021, 143 (34), 13525–13537. 10.1021/jacs.1c02287. 34398608

[ref46] Frenkel-Pinter M.; Haynes J. W.; Mohyeldin A. M.; C M.; Sargon A. B.; Petrov A. S.; Krishnamurthy R.; Hud N. V.; Williams L. D.; Leman L. J. Mutually Stabilizing Interactions between Proto-Peptides and RNA. Nat. Commun. 2020, 11 (1), 3137 10.1038/s41467-020-16891-5. 32561731 PMC7305224

[ref47] Chandru K.; Guttenberg N.; Giri C.; Hongo Y.; Butch C.; Mamajanov I.; Cleaves H. J. Simple Prebiotic Synthesis of High Diversity Dynamic Combinatorial Polyester Libraries. Commun. Chem. 2018, 10.1038/s42004-018-0031-1.

[ref48] Jia T. Z.; Chandru K.; Hongo Y.; Afrin R.; Usui T.; Myojo K.; Cleaves H. J. 2nd. Membraneless Polyester Microdroplets as Primordial Compartments at the Origins of Life.. Proc. Natl. Acad. Sci. U. S. A. 2019, 116 (32), 15830–15835. 10.1073/pnas.1902336116. 31332006 PMC6690027

[ref49] Chen C.; Yi R.; Igisu M.; Sakaguchi C.; Afrin R.; Potiszil C.; Kunihiro T.; Kobayashi K.; Nakamura E.; Ueno Y.; Antunes A.; Wang A.; Chandru K.; Hao J.; Jia T. Z. Spectroscopic and Biophysical Methods to Determine Differential Salt-Uptake by Primitive Membraneless Polyester Microdroplets. Small Methods 2023, 7 (12), e2300119 10.1002/smtd.202300119. 37203261

[ref50] Brangwynne C. P.; Eckmann C. R.; Courson D. S.; Rybarska A.; Hoege C.; Gharakhani J.; Jülicher F.; Hyman A. A. Germline P Granules Are Liquid Droplets That Localize by Controlled Dissolution/condensation. Science 2009, 324 (5935), 1729–1732. 10.1126/science.1172046. 19460965

[ref51] Chen C.; Yamanaka Y.; Ueda K.; Li P.; Miyagi T.; et al. Phase Separation and Toxicity of C9orf72 Poly (PR) Depends on Alternate Distribution of Arginine. J. Cell Biol. 2021, 220 (11), e202103160 10.1083/jcb.202103160. 34499080 PMC8438627

[ref52] Jia T. Z.; Bapat N. V.; Verma A.; Mamajanov I.; Cleaves H. J. 2nd; Chandru K. Incorporation of Basic α-Hydroxy Acid Residues into Primitive Polyester Microdroplets for RNA Segregation. Biomacromolecules 2021, 22 (4), 1484–1493. 10.1021/acs.biomac.0c01697. 33663210

[ref53] Poddar A.; Satthiyasilan N.; Wang P.-H.; Chen C.; Yi R.; Chandru K.; Jia T. Z. Reactions Driven by Primitive Nonbiological Polyesters. Acc. Chem. Res. 2024, 57 (15), 2048–2057. 10.1021/acs.accounts.4c00167. 39013010

[ref54] Monnard P.-A.; Walde P. Current Ideas about Prebiological Compartmentalization. Life 2015, 5 (2), 1239–1263. 10.3390/life5021239. 25867709 PMC4500137

[ref55] Ichihashi N.; Yomo T. Positive Roles of Compartmentalization in Internal Reactions. Curr. Opin. Chem. Biol. 2014, 22, 12–17. 10.1016/j.cbpa.2014.06.011. 25032508

[ref56] Afrin R.; Chen C.; Sarpa D.; Sithamparam M.; et al. The Effects of Dehydration Temperature and Monomer Chirality on Primitive Polyester Synthesis and Microdroplet Assembly. Macromol. Chem. Phys. 2022, 223, 2200235 10.1002/macp.202200235.

[ref57] Chandru K.; Jia T. Z.; Mamajanov I.; Bapat N.; Cleaves H. J. 2nd. Prebiotic Oligomerization and Self-Assembly of Structurally Diverse Xenobiological Monomers. Sci. Rep. 2020, 10 (1), 17560 10.1038/s41598-020-74223-5. 33067516 PMC7567815

[ref58] Ruiz-Bermejo M.; Osuna-Esteban S.; Zorzano M.-P. Role of Ferrocyanides in the Prebiotic Synthesis of α-Amino Acids. Orig Life Evol Biosph 2013, 43 (3), 191–206. 10.1007/s11084-013-9336-3. 23780697

[ref59] Miller S. L. The Atmosphere of the Primitive Earth and the Prebiotic Synthesis of Amino Acids. Origins Life Evol Biosphere 1974, 5 (1–2), 139–151. 10.1007/BF00927019. 4842068

[ref60] Miyazaki Y.; Sawano M.; Kawamura K. Low-Molecular-Weight Hydroxyacids in Marine Atmospheric Aerosol: Evidence of a Marine Microbial Origin. Biogeosciences 2014, 11, 4407 10.5194/bg-11-4407-2014.

[ref61] Du R.; Yang D.; Jiang G.; Song Y.; Yin X. An Approach for In Situ Rapid Detection of Deep-Sea Aromatic Amino Acids Using Laser-Induced Fluorescence. Sensors 2020, 20 (5), 1330 10.3390/s20051330. 32121409 PMC7085558

[ref62] Masamba W. Petasis vs. Strecker Amino Acid Synthesis: Convergence, Divergence and Opportunities in Organic Synthesis. Molecules 2021, 26 (6), 1707 10.3390/molecules26061707. 33803879 PMC8003338

[ref63] Gleißner P. The Earth–Moon Late-Accretion Conundrum. Nat. Geosci. 2019, 12 (9), 683–684. 10.1038/s41561-019-0445-0.

[ref64] Harrison T. M. The Lunar Surface and Late Heavy Bombardment Concept. In Hadean Earth; Harrison T. M., Ed.; Springer, 2020; pp 59–100.

[ref65] Pizzarello S.; Schrader D. L.; Monroe A. A.; Lauretta D. S. Large Enantiomeric Excesses in Primitive Meteorites and the Diverse Effects of Water in Cosmochemical Evolution. Proc. Natl. Acad. Sci. U. S. A. 2012, 109 (30), 11949–11954. 10.1073/pnas.1204865109. 22778439 PMC3409747

[ref66] Aponte J. C.; Elsila J. E.; Hein J. E.; Dworkin J. P.; Glavin D. P.; McLain H. L.; Parker E. T.; Cao T.; Berger E. L.; Burton A. S. Analysis of Amino Acids, Hydroxy Acids, and Amines in CR Chondrites. Meteorit. Planet. Sci. 2020, 55 (11), 2422–2439. 10.1111/maps.13586. 33536738 PMC7839561

[ref67] Jerabek-Willemsen M.; Wienken C. J.; Braun D.; Baaske P.; Duhr S. Molecular Interaction Studies Using Microscale Thermophoresis. Assay Drug Dev. Technol. 2011, 9 (4), 342–353. 10.1089/adt.2011.0380. 21812660 PMC3148787

[ref68] Dietrich V. J.; Lagios E.; Reusser E.; Sakkas V.; Gartzos E.; Kyriakopoulos K. The Enigmatic Zerelia Twin-Lakes (Thessaly, Central Greece): Two Potential Meteorite Impact Craters. Solid Earth Discuss. 2013, 5, 1511–1573.

[ref69] Branney M.; Acocella V. Chapter 16 - Calderas. In The Encyclopedia of Volcanoes, 2nd ed.; Sigurdsson H., Ed.; Academic Press, 2015; pp 299–315.

[ref70] Hadding A. The First Rains and Their Geological Significance. GFF. 1929, 51 (1), 19–29. 10.1080/11035892909447052.

[ref71] Lunine J. I. Physical Conditions on the Early Earth. Philos. Trans. R. Soc. London B Biol. Sci. 2006, 361 (1474), 1721–1731. 10.1098/rstb.2006.1900. 17008213 PMC1664683

[ref72] Kreysing M.; Keil L.; Lanzmich S.; Braun D. Heat Flux across an Open Pore Enables the Continuous Replication and Selection of Oligonucleotides towards Increasing Length. Nat. Chem. 2015, 7 (3), 203–208. 10.1038/nchem.2155. 25698328

[ref73] Yi R.; Jia T. Z.; Meringer M.; Marshall L. K.; Chen C.; McGlynn S. E.; Fahrenbach A. C.; Cleaves H. J. 2nd. Alternating Co-Synthesis of Glycol Nucleic Acid (GNA) Monomers with Dicarboxylic Acids via Drying. Chem. Commun. 2023, 59 (45), 6865–6868. 10.1039/D2CC06818D. 37195424

[ref74] Ianeselli A.; Tetiker D.; Stein J.; Kühnlein A.; Mast C. B.; Braun D.; Dora Tang T.-Y. Non-Equilibrium Conditions inside Rock Pores Drive Fission, Maintenance and Selection of Coacervate Protocells. Nat. Chem. 2022, 14 (1), 32–39. 10.1038/s41557-021-00830-y. 34873298 PMC8755537

[ref75] Ranga U. SARS-CoV-2 Aerosol and Droplets: An Overview. Virusdisease 2021, 32 (2), 190–197. 10.1007/s13337-021-00660-z. 33907703 PMC8061877

[ref76] Allfrey V. G.; Meudt R.; Hopkins J. W.; Mirsky A. E. Sodium-Dependent “Transport” Reactions in the Cell Nucleus and Their Role in Protein and Nucleic Acid Synthesis. Proc. Natl. Acad. Sci. U. S. A. 1961, 47 (7), 907–932. 10.1073/pnas.47.7.907. 13682569 PMC221304

[ref77] Marakhova I.; Domnina A.; Shatrova A.; Borodkina A.; Burova E.; Pugovkina N.; Zemelko V.; Nikolsky N. Proliferation-Related Changes in K^+^ Content in Human Mesenchymal Stem Cells. Sci. Rep. 2019, 9 (1), 346 10.1038/s41598-018-36922-y. 30674973 PMC6344592

[ref78] Yamagami R.; Sieg J. P.; Bevilacqua P. C. Functional Roles of Chelated Magnesium Ions in RNA Folding and Function. Biochemistry 2021, 60 (31), 2374–2386. 10.1021/acs.biochem.1c00012. 34319696 PMC8747768

[ref79] Xu N. On the Concept of Resting Potential—Pumping Ratio of the Na^+^/K^+^ Pump and Concentration Ratios of Potassium Ions Outside and Inside the Cell to Sodium Ions Inside and Outside the Cell. J. Membr. Biol. 2013, 246 (1), 75–90. 10.1007/s00232-012-9507-6. 23262466

[ref80] Model M. A.; Mahajan A.; Düsterwald K. M.; Dmitriev A. V. Nucleic Acids Regulate Intracellular Ions and Membrane Potential. Paracelsus Proc. Exp. Med. 2023, 2 (S1), 13–29. 10.33594/000000613.

[ref81] De Mel J. U.; Gupta S.; Perera R. M.; Ngo L.; Zolnierczuk P.; Bleuel M.; Pingali S. V.; Schneider G. J. Influence of External NaCl Salt on Membrane Rigidity of Neutral DOPC Vesicles. Langmuir 2020, 36 (32), 9356–9367. 10.1021/acs.langmuir.0c01004. 32672981

[ref82] Tagami S.; Li P. The Origin of Life: RNA and Protein Co-Evolution on the Ancient Earth. Dev. Growth Differ. 2023, 65 (3), 167–174. 10.1111/dgd.12845. 36762966

[ref83] Rowan M. G. Passive-Margin Salt Basins: Hyperextension, Evaporite Deposition, and Salt Tectonics. Basin Res. 2014, 26 (1), 154–182. 10.1111/bre.12043.

[ref84] Hovland M.; Rueslåtten H.; Johnsen H. K. Salt Formation, Accumulation, and Expulsion Processes During Ocean Rifting—New Insight Gained from the Red Sea. In Geological Setting, Palaeoenvironment and Archaeology of the Red Sea; Rasul N. M. A., Stewart I. C. F., Eds.; Springer, 2019; pp 233–257.

[ref85] Olson S.; Jansen M. F.; Abbot D. S.; Halevy I.; Goldblatt C. The Effect of Ocean Salinity on Climate and Its Implications for Earth’s Habitability. Geophys. Res. Lett. 2022, 49 (10), e2021GL095748 10.1029/2021GL095748. PMC9286645 35864818

[ref86] Mourelle M. L.; Gómez C. P.; Legido J. L. Cosmeceuticals and Thalassotherapy: Recovering the Skin and Well-Being after Cancer Therapies. NATO Adv. Sci. Inst. Ser. E Appl. Sci. 2023, 13 (2), 850 10.3390/app13020850.

[ref87] Galvis-Sánchez A. C.; Lopes J. A.; Delgadillo I.; Rangel A. O. S. S. Sea Salt. In Food Protected Designation of Origin - Methodologies and Applications; Comprehensive analytical chemistry; Elsevier, 2013; Vol. 60, pp 719–740.

[ref88] Foriel J.; Philippot P.; Rey P.; Somogyi A.; Banks D.; Ménez B. Biological Control of Cl/Br and Low Sulfate Concentration in a 3.5-Gyr-Old Seawater from North Pole, Western Australia. Earth Planet. Sci. Lett. 2004, 228 (3–4), 451–463. 10.1016/j.epsl.2004.09.034.

[ref89] Jones C.; Nomosatryo S.; Crowe S. A.; Bjerrum C. J.; Canfield D. E. Iron oxides, divalent cations, silica, and the early earth phosphorus crisis. Geology 2015, 43 (2), 135–138. 10.1130/G36044.1.

[ref90] Katz A.; Starinsky A. Geochemical History of the Dead Sea. Aquat. Geochem. 2009, 15 (1–2), 159–194. 10.1007/s10498-008-9045-0.

[ref91] Antunes A.; Ngugi D. K.; Stingl U. Microbiology of the Red Sea (and Other) Deep-Sea Anoxic Brine Lakes. Environ. Microbiol. Rep. 2011, 3 (4), 416–433. 10.1111/j.1758-2229.2011.00264.x. 23761304

[ref92] Hansma H. G. Potassium at the Origins of Life: Did Biology Emerge from Biotite in Micaceous Clay?. Life 2022, 12 (2), 301 10.3390/life12020301. 35207588 PMC8880093

[ref93] Remko M.; Rode B. M. Effect of Metal Ions (Li^+^, Na^+^, K^+^, Mg^2+^, Ca^2+^, Ni^2+^, Cu^2+^, and Zn^2+^) and Water Coordination on the Structure of Glycine and Zwitterionic Glycine. J. Phys. Chem. A 2006, 110 (5), 1960–1967. 10.1021/jp054119b. 16451030

[ref94] Ozgurel O.; Mousis O.; Pauzat F.; Ellinger Y.; Markovits A.; Vance S.; Leblanc F. Sodium, Potassium, and Calcium in Europa: An Atomic Journey through Water Ice. Astrophys. J. Letters 2018, 865 (2), L16 10.3847/2041-8213/aae091.

[ref95] Trumbo S. K.; Brown M. E.; Hand K. P. Sodium Chloride on the Surface of Europa. Sci. Adv. 2019, 5 (6), eaaw7123 10.1126/sciadv.aaw7123. 31206026 PMC6561749

[ref96] Primm K. M.; Stillman D. E.; Michaels T. I. Investigating the Hysteretic Behavior of Mars-Relevant Chlorides. Icarus 2020, 342, 113342 10.1016/j.icarus.2019.06.003.

[ref97] Vidal-Madjar A.; Huitson C. M.; Bourrier V.; Désert J.-M.; Ballester G.; Lecavelier des Etangs A.; Sing D. K.; Ehrenreich D.; Ferlet R.; Hébrard G.; McConnell J. C. Magnesium in the Atmosphere of the Planet HD 209458 B: Observations of the Thermosphere-Exosphere Transition Region. Astron. Astrophys. 2013, 560, A54 10.1051/0004-6361/201322234.

[ref98] Mernagh T. P.; Bastrakov E. N.; Jaireth S.; de Caritat P.; English P. M.; Clarke J. D. A. A Review of Australian Salt Lakes and Associated Mineral Systems. Aust. J. Earth Sci. 2016, 63 (2), 131–157. 10.1080/08120099.2016.1149517.

[ref99] Kaiser J.; Ön B.; Arz H. W.; Akçer-Ön S. Sedimentary Lipid Biomarkers in the Magnesium Rich and Highly Alkaline Lake Salda (south-Western Anatolia). J. Limnol. 2016, 10.4081/jlimnol.2016.1337.

