# Supplementary material for: Probing the Limits of Reactant Concentration and Volume in Primitive Polyphenyllactate Synthesis and Microdroplet Assembly Processes
Source: ACS Bio Med Chem Au. 2025 Jan 9;5(1):131–42. doi: 10.1021/acsbiomedchemau.4c00082 (PMC11843335; doi:10.1021/acsbiomedchemau.4c00082)
Supplement: Supplementary file 1 — bg4c00082_si_001.pdf [file bg4c00082_si_001.pdf]

Supporting Information

Probing the limits of reactant concentration and volume in primitive polyphenyllactate synthesis and microdroplet assembly processes

Mahendran Sithamparam[1], Rehana Afrin[2], Navaniswaran Tharumen[1], Ming-Jing He[3], Chen Chen[4], Ruiqin Yi[5], Po-Hsiang Wang[3,6], Tony Z. Jia[2,7,*], Kuhan Chandru[1,8,9,*]

[1]Space Science Center (ANGKASA), Institute of Climate Change, National University of Malaysia, Selangor, 43650 Malaysia
[2]Earth-Life Science Institute, Institute of Future Science, Institute of Science Tokyo, 2-12-1-IE-1 Ookayama, Meguro-ku, Tokyo 152-8550, Japan
[3]Department of Chemical Engineering and Materials Engineering, National Central University, No. 300, Zhongda Rd., Zhongli District, Taoyuan 32001, Taiwan (R.O.C.)
[4]Biofunctional Catalyst Research Team, RIKEN Center for Sustainable Resource Science (CSRS), 2-1 Hirosawa, Wako, Saitama 351-0198, Japan
[5]State Key Laboratory of Isotope Geochemistry and CAS Center for Excellence in Deep Earth Science, Guangzhou Institute of Geochemistry, Chinese Academy of Sciences, Guangzhou, 510640, China
[6]Graduate Institute of Environmental Engineering, National Central University, No. 300, Zhongda Road, Zhongli District, Taoyuan City 320, Taiwan
[7]Blue Marble Space Institute of Science, 600 1st Ave, Floor 1, Seattle, WA 98104, USA
[8]Polymer Research Center (PORCE), Faculty of Science and Technology, National University of Malaysia, Selangor, 43600 Malaysia
[9]Institute of Physical Chemistry, CENIDE, University of Duisburg-Essen, 45141 Essen, Germany

***Corresponding Authors:**
Kuhan Chandru
Space Science Center (ANGKASA), Institute of Climate Change, National University of Malaysia
Selangor, 43650 Malaysia
Email: kuhan@ukm.edu.my

Tony Z. Jia
Earth-Life Science Institute, Institute of Future Science, Institute of Science Tokyo
2-12-1-IE-1 Ookayama, Meguro-ku, Tokyo 152-8550, Japan
Email: tzjia@elsi.jp

Table of Contents

Description of Data Files	3
Supplementary Method	4
Supplementary Figures	4
Supplementary Tables	9

Description of Files

Raw MALDI Spectra

"MALDI_Volume_Variation": Raw Spectra for reaction initial volume variation experiments

"MALDI_Concentration_Variation": Raw Spectra for reactant concentration variation experiments

"MALDI_Salt_Solution_Variation": Raw Spectra for reaction in 1 M salt variation experiments

MALDI Peaklists

"Peaklists_Volume_Variation": Peaklists for reaction initial volume variation experiments

"Peaklists_Concentration_Variation": Peaklists for reactant concentration variation experiments

"Peaklists_Salt_Solution_Variation": Peaklists for reaction in 1 M salt variation experiments

Microscopy Images

"Images_Volume_Variation": Microscopy images for volume variation experiments

"Images_Concentration_Variation": Microscopy images for volume variation experiments

"Images_Salt_Solution_Variation": Microscopy images for reaction in 1 M salt variation experiments

"Images_Control": Microscopy images of control reactions

Supplementary Method

Droplet Size Image Analysis. For image analysis using FIJI, default thresholding was used (dark background was selected if necessary), in addition to the “Analyze Particles” function with Size = (1.05 – infinity) and Circularity = 0.00 – 1.00, while excluding any particles “detected” that reside on the edge of the image (except in the case of the $MgCl_2$ sample where there was no MMD assembly observed). Droplets that were smaller than 10 square µm (which could be errors and not actually particles due to overexposure, etc.; this is the reason that there are no detected particles less than 3.5 µm in diameter), and large areas that were clearly not droplets (which are all caused by patches of heterogeneous background intensity typically at the edges of the image that were incorrectly identified as particles) were also removed. We assumed that all particles were spheres that were captured at their respective equators, and thus back-calculated the diameter of each particle from its computed area.

Supplementary Figures

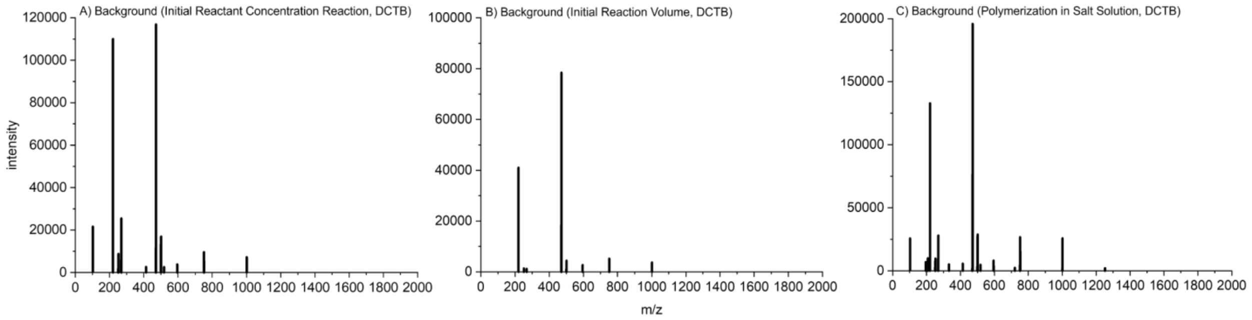

Figure S1. Representative MALDI-ToF-MS background spectra derived using the DCTB matrix. Background spectra were obtained separately for each set of experimental conditions. A) Background MS spectrum for the set of experiments probing differing initial PA concentration (**Fig. 1**), B) Background MS spectrum for the set of experiments probing differing initial PA volume (**Fig. 2**), C) Background MS spectrum for the set of experiments probing PA polymerization in 1 M of NaCl, KCl, and $MgCl_2$ (**Fig. 3**).

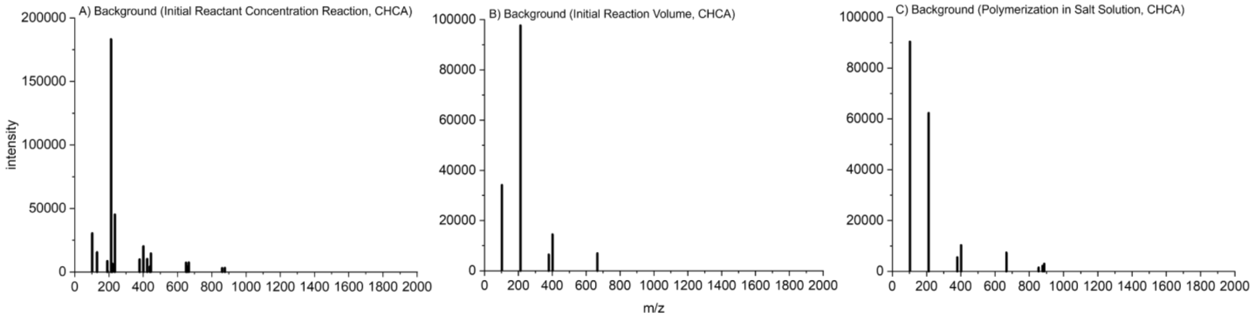

Figure S2. Representative MALDI-ToF-MS background spectra derived using the CHCA matrix. Background spectra were obtained separately for each set of experimental conditions. A) Background MS spectrum for the set of experiments probing differing initial PA concentration (**Fig. 1**), B) Background MS spectrum for the set of experiments probing differing initial PA volume (**Fig. 2**), C) Background MS spectrum for the set of experiments probing PA polymerization in 1 M of NaCl, KCl, or $MgCl_2$ (**Fig. 3**).

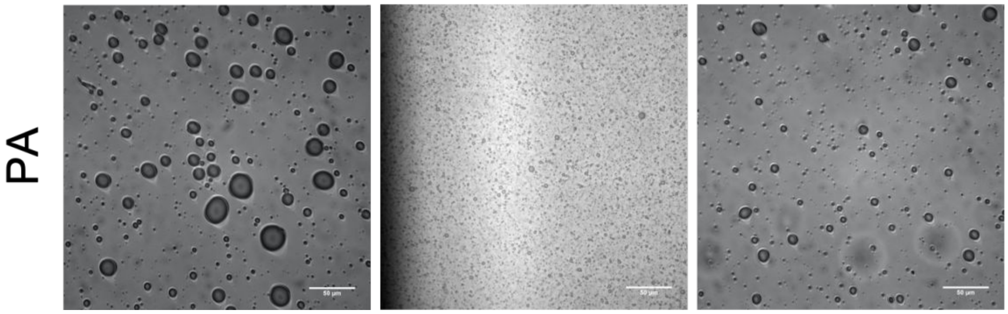

Figure S3: Representative control microscopy images of products from PA polymerization reactions (500 mM reactant concentration and 500 μL reaction volume) following rehydration. Scale bars: 50 μm.

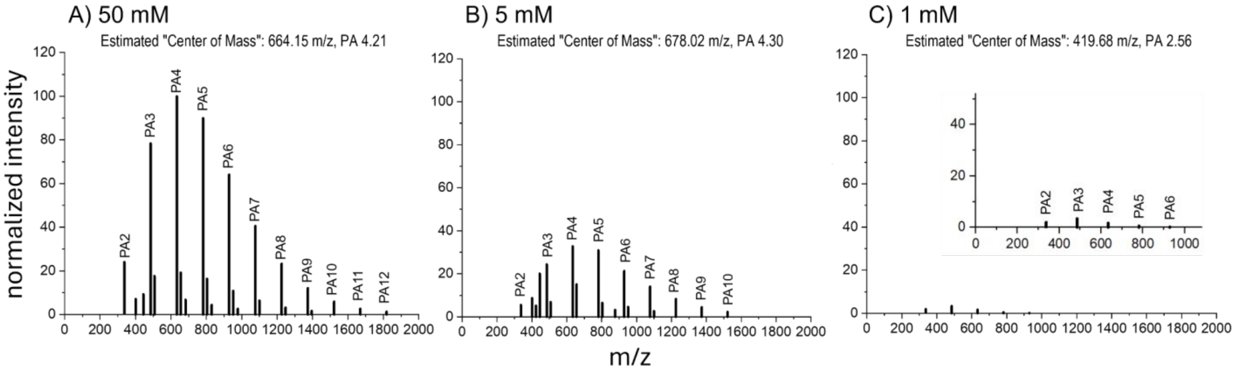

Figure S4: Representative MALDI-ToF-MS spectra of products from variable initial reactant concentration ((A) 50 mM, (B) 5 mM, and (C) 1 mM) PA polymerization reactions (at a constant 500 μL reactant volume). Labeled peaks are sodiated ($M+Na^+$). The spectra shown here were derived using the CHCA matrix. The peaks were normalized based on the intensity of the highest peak observed across all conditions in the same set of experiments using the same matrix; in this set of spectra, the highest peak is from the 50 mM spectrum.

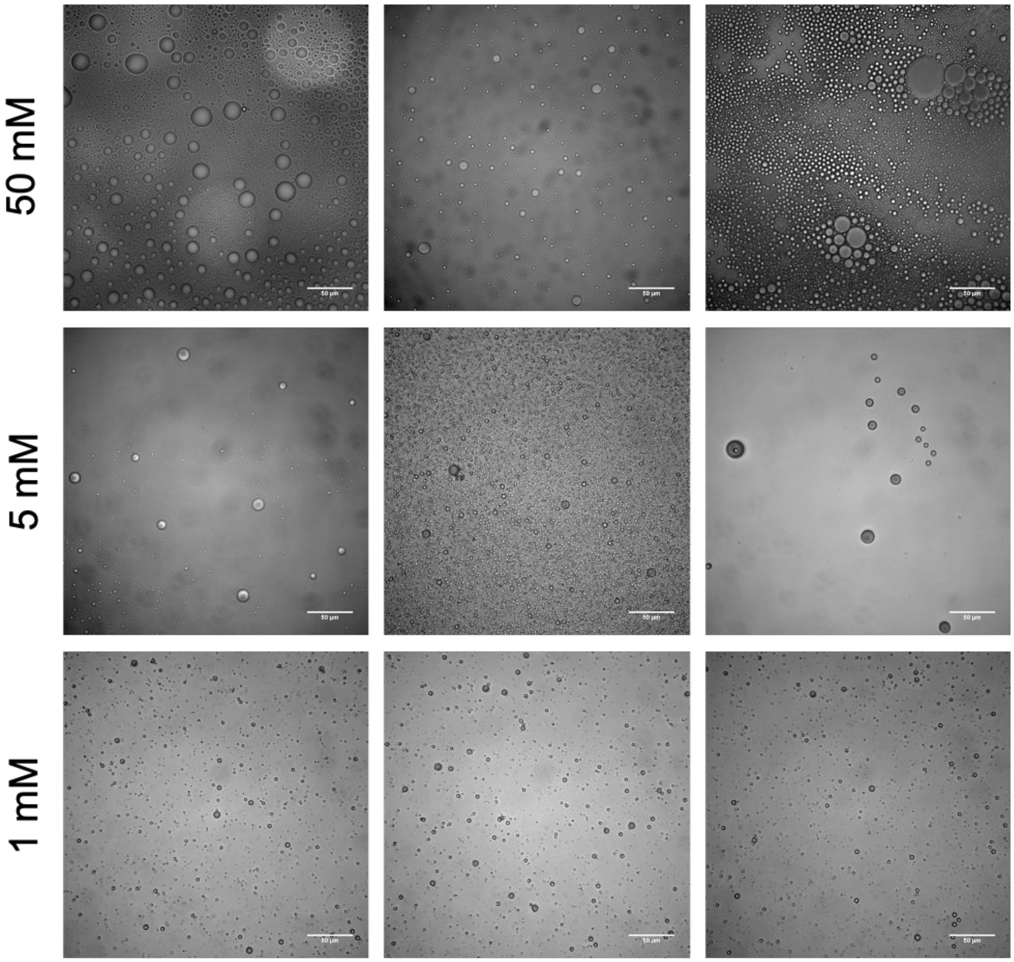

Figure S5. Representative microscopy images of products from variable initial (50 mM, 5 mM, and 1 mM) concentration PA polymerization reactions (at constant 500 µL reaction volume) following rehydration. Three different images of each sample are reported to show reproducibility. Scale bars: 50 µm.

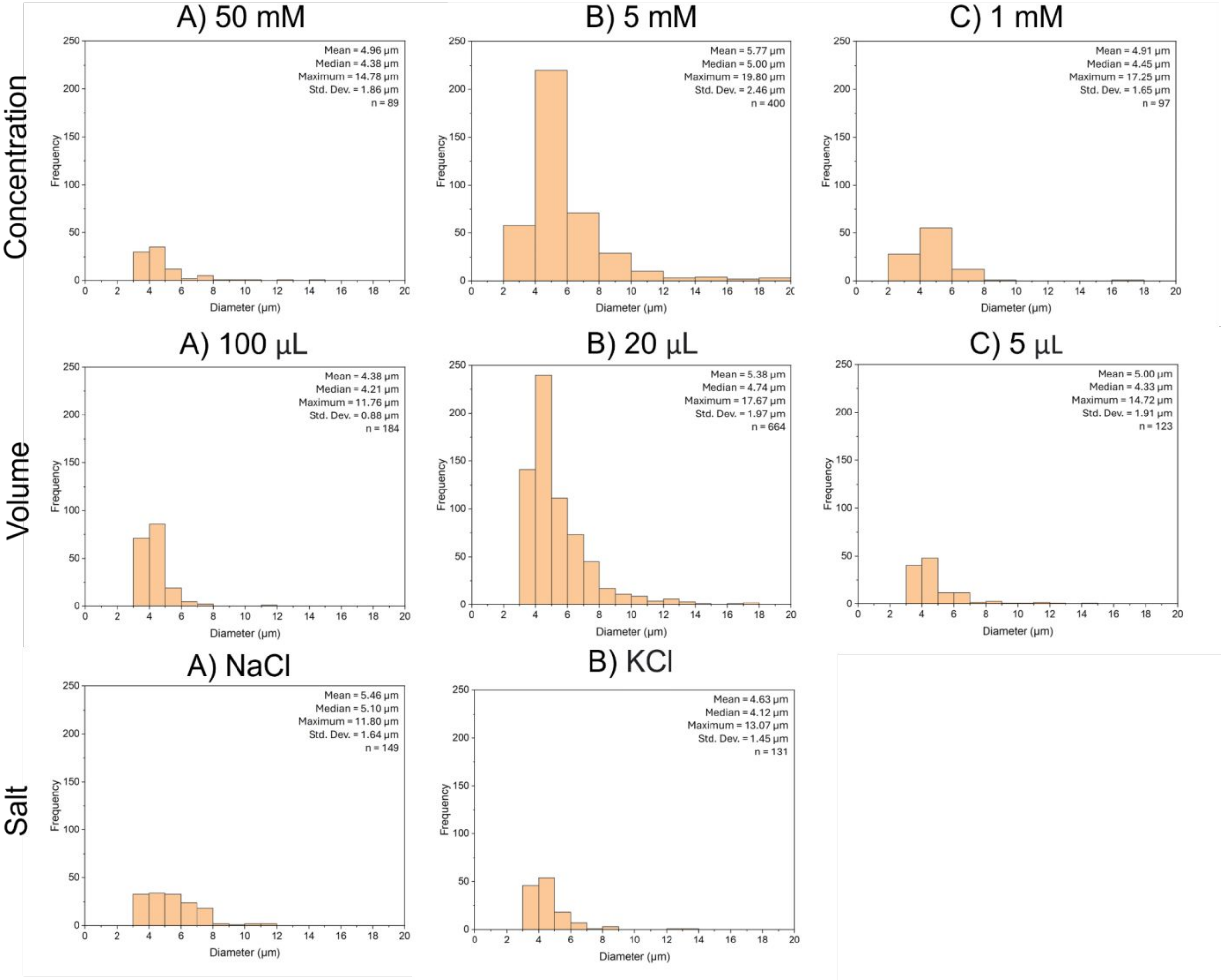

Figure S6. Histograms showing size distributions of the spherical particles (diameter in µm) from each image in Figs. 1–3 (except the image obtained from $MgCl_2$, which did not show any MMD assembly).

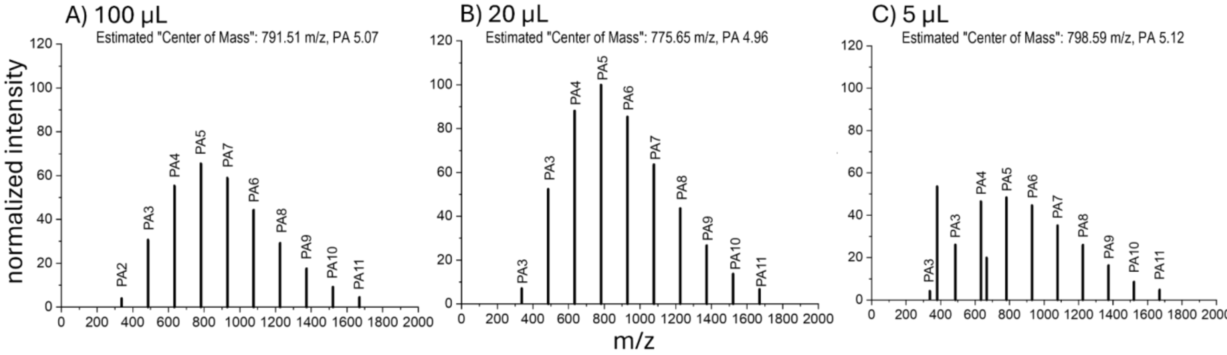

Figure S7: Representative MALDI-ToF-MS spectra of products from variable initial reaction volume (A) 100 µL, B) 20 µL, and C) 5 µL) PA polymerization reactions (at a constant 500 nM reactant Volume). Labeled peaks are sodiated ($M+Na^+$). The spectra shown here were derived using the CHCA matrix. The peaks were normalized based on the intensity of the highest peak observed across all conditions in the same set of experiments using the same matrix; in this set of spectra, the highest peak is from the 20 µL spectrum.

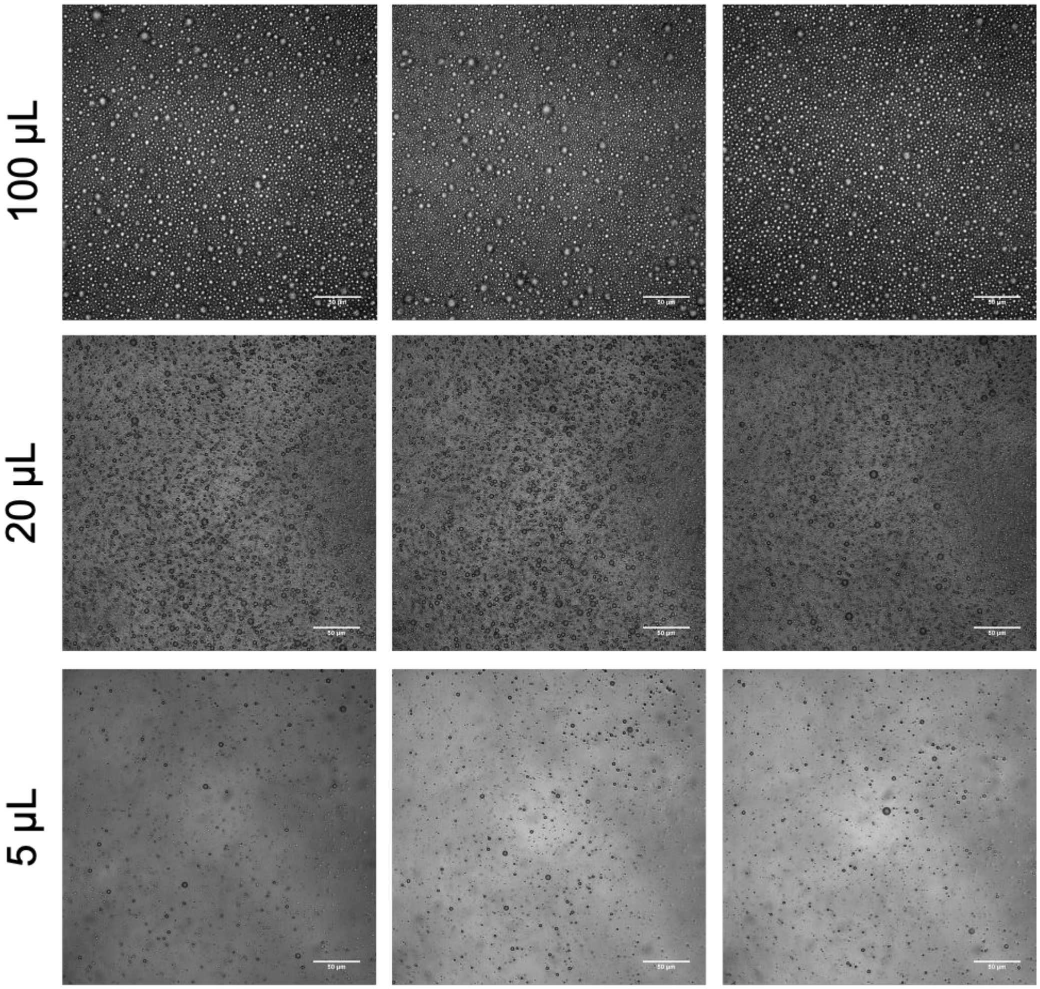

Figure S8. Representative microscopy images of products from variable volume (100μL, 20μL, and 5μL) PA polymerization reactions (at constant 500 mM reactant concentration) following rehydration. Three different images of each sample are reported to show reproducibility. Scale bars: 50 μm.

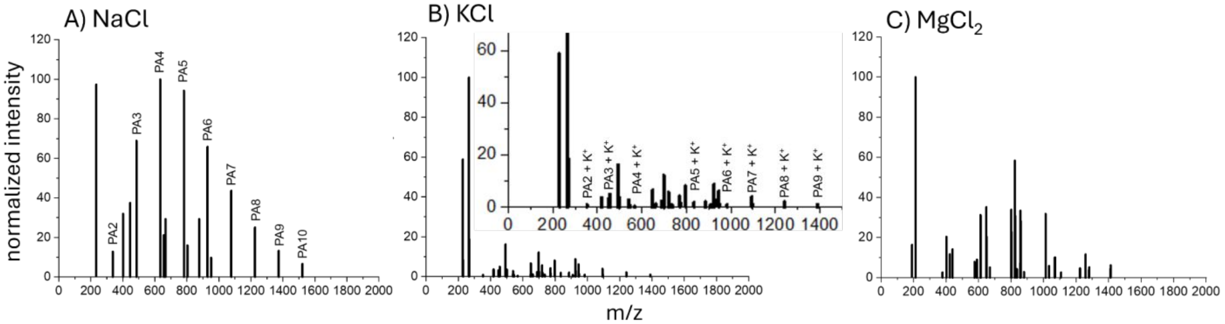

Figure S9: Representative MALDI-ToF-MS spectra of products from PA polymerization reactions in the presence of various salts A) NaCl, B) KCl, and C) $MgCl_2$ (at a constant 1 M). Labeled peaks are sodiated ($M+Na^+$) or potassiated ($M+K^+$) (inset). We did not observe any clear PA polymerization in the $MgCl_2$-containing reaction. The spectra shown here were derived using the CHCA matrix. The peaks were normalized based on the intensity of the highest peak (not necessarily a polymer product peak) observed from each spectra separately.

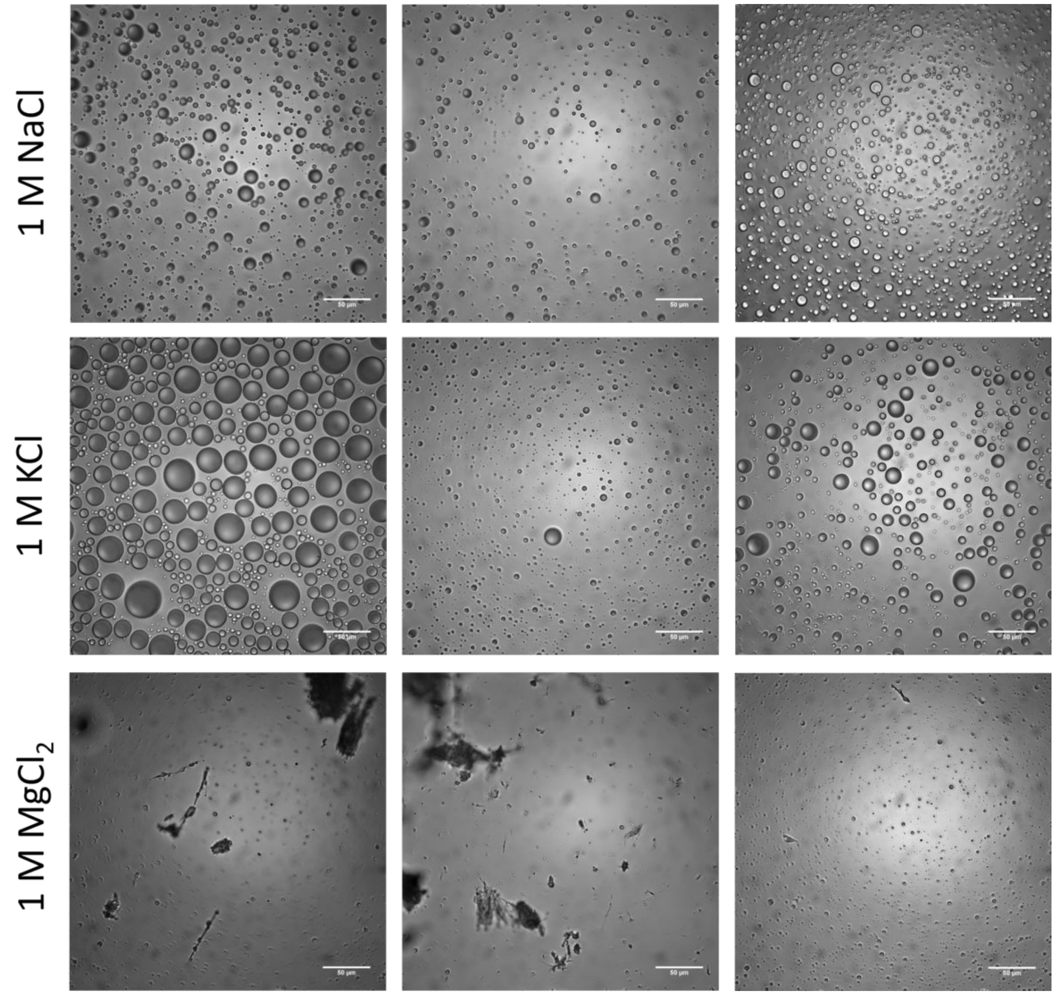

Figure S10. Representative microscopy images of products from 500 mM PA polymerization reactions (at constant 500 µL reaction volume) in the presence of 1 M salts following rehydration. Three different images of each sample are reported to show reproducibility. Scale bars: 50 µm.

Supplementary Tables

Table S1. Summarized details of previously tested experimental conditions.

Title	Details of tested conditions	Link
Simple prebiotic synthesis of high diversity dynamic combinatorial polyester libraries	Up to five αHAs (LA, MA, SA, GA and PA) were reacted simultaneously.<br><br>**Reactants**: Individual GA and LA, mixed GA+LA, and five mixed αHAs (LA, MA, SA, GA and PA)<br>**Reaction temperature**: 60–100 °C<br>**Duration**: 24 hrs<br>**Initial volume of reaction**: 500 µL<br>**Total concentration of reactants**: 0.15 M	https://www.nature.com/articles/s42004-018-0031-1#additional-information

Membraneless polyester microdroplets as primordial compartments at the origins of life	Up to five αHAs (LA, MA, SA, GA, and PA) were reacted simultaneously.<br><br>**Reactants**: Individual and combinations of mixtures of two, three, four, and five αHAs<br>**Reaction temperature**: 80°C<br>**Duration**: 1 week<br>**Initial volume of reaction**: 500 µL<br>**Total concentration of reactants**: 500 mM	https://www.pnas.org/doi/10.1073/pnas.1902336116
Prebiotic oligomerization and self-assembly of structurally diverse xenobiological monomers	Small ring monomers (DO, LD, GD, CN, CM, VN, 2OX, MM, TB, MO, 1MOX, GA, LA) and Gly were reacted pairwise at most.<br><br>**Reactants**: Individual monomers and two monomers mixed (Gly+other ring monomers)<br>**Reaction temperature**: 60–100°C<br>Duration: 24 hrs<br>**Initial volume of reaction**: 100 µL (individual) and 200 µL (mixed)<br>**Total concentration of reactants**: 1 M	https://www.nature.com/articles/s41598-020-74223-5#Sec8
Incorporation of Basic α-Hydroxy Acid Residues into Primitive Polyester Microdroplets for RNA Segregation	4a2h was reacted with GA, LA, PA, SA or MA.<br><br>**Reactants**: Up to two αHAs.<br>**Reaction temperature**: 80°C<br>**Duration**: 1 week<br>**Initial volume of reaction**: 500 µL<br>**Total concentration of reactants**: 500 mM	https://pubs.acs.org/doi/10.1021/acs.biomac.0c01697
The Effects of Dehydration Temperature and Monomer Chirality on Primitive Polyester Synthesis and Microdroplet Assembly	Dehydration reactions at varying temperatures (40–150°C) and monomer chirality D,L, and DL (LA and PA) were performed.<br><br>**Reactants**: Individual LA and PA of varying chirality<br>**Reaction temperature**: 40–150°C<br>**Duration**: Varies depending on synthesis temperature<br>**Initial volume of reaction**: 500 µL<br>**Total concentration of reactant**: 500 mM	https://onlinelibrary.wiley.com/doi/full/10.1002/macp.202200235
Spectroscopic and Biophysical Methods to Determine Differential Salt-Uptake by Primitive Membraneless Polyester Microdroplets	PA was subjected to dehydration synthesis in 1) up to 100 mM salts; 2) incorporated with Malic or 4a2h.<br><br>**Reactants**: PA, malic acid, and 4a2h, up to two components per reaction<br>**Salts**: NaCl, KCl, $MgCl_2$ and $CaCl_2$<br>**Reaction temperature**: 80°C<br>**Duration**: 1 week<br>**Initial volume of reaction**: 500 µL<br>**Total concentration of reactant**: 500 mM<br>**Concentration of each salt**: 10 mM and 100 mM	https://onlinelibrary.wiley.com/doi/full/10.1002/smtd.202300119

*Glycolic acid (GA), Lactic acid (LA), 2-hydroxy-4-methylpentanoic acid (MA), 2-hydroxy-4-(methylsulfanyl)butanoic acid (SA), 3-phenyllactic acid (PA), 1,4-Dioxan-2-one (DO), Lactide (LD), Glycolide (GD), ε-Caprolactone (CN), ε-Caprolactam (CM),

δ-Valerolactone (VN), 2-Oxopiperazine (2OX), 4-Methylmorpholin-2-one (MM), γ-Thiobutyrolactone (TB), Morpholine-2-one (MO), 1-Methyl-3-oxopiperazine (1MOX), 4-amino-2-hydroxybutyric acid (4a2h), Malic acid (Malic), and Glycine (Gly)

Table S2. Peaklist sorted by increasing mass for PA polymer produced from a reaction with a starting PA monomer concentration of 50 mM using DCTB matrix at a constant 500 μL initial reaction volume. Spectrum is shown in Figure 1.

Observed Mass (Da)	Intensity	Calculated Mass (Da)	Identity	Adduct	Error (PPM)
337.1305	4085.4167	337.1046	PA2	$M+Na^+$	76.6191
485.1904	22930.9616	485.1571	PA3	$M+Na^+$	68.7142
633.2444	52098.6226	633.2095	PA4	$M+Na^+$	55.1078
781.3022	74906.6752	781.2619	PA5	$M+Na^+$	51.5410
929.3551	71841.4761	929.3143	PA6	$M+Na^+$	43.8566
1077.4149	55357.2748	1077.3668	PA7	$M+Na^+$	44.6858
1225.4684	36138.8473	1225.4192	PA8	$M+Na^+$	40.1347
1373.5289	20640.8194	1373.4716	PA9	$M+Na^+$	41.7196
1521.5769	10534.7281	1521.5241	PA10	$M+Na^+$	34.7325
1669.6329	5111.7292	1669.5765	PA11	$M+Na^+$	33.8101
1817.6945	2476.4962	1817.6289	PA12	$M+Na^+$	36.0602
1965.7483	1129.6089	1965.6813	PA13	$M+Na^+$	34.0589

Table S3. Peaklist sorted by increasing mass for PA polymers produced from a reaction with a starting PA monomer concentration of 5 mM using DCTB matrix at a constant 500 μL initial reaction volume. Spectrum is shown in Figure 1.

Observed Mass (Da)	Intensity	Calculated Mass (Da)	Identity	Adduct	Error (PPM)
337.1351	1950.7303	337.1046	PA2	$M+Na^+$	90.4699
485.1904	13586.5393	485.1571	PA3	$M+Na^+$	68.7142

633.2508	25823.2556	633.2095	PA4	M+Na$^+$	65.2287
781.3022	35230.6772	781.2619	PA5	M+Na$^+$	51.5410
929.3629	32534.1740	929.3143	PA6	M+Na$^+$	52.1976
1077.4149	26818.8313	1077.3668	PA7	M+Na$^+$	44.6858
1225.4773	19163.6583	1225.4192	PA8	M+Na$^+$	47.4066
1373.5289	12239.3616	1373.4716	PA9	M+Na$^+$	41.7196
1521.5869	7198.4063	1521.5241	PA10	M+Na$^+$	41.3112
1669.6433	4101.8197	1669.5765	PA11	M+Na$^+$	40.0249
1817.7053	2401.9758	1817.6289	PA12	M+Na$^+$	42.0373
1965.7596	1184.7414	1965.6813	PA13	M+Na$^+$	39.8343

Table S4. Peaklist sorted by increasing mass for PA polymers produced from a reaction with a starting PA monomer concentration of 1 mM using DCTB matrix at a constant 500 µL initial reaction volume. Spectrum is shown in Figure 1.

Observed Mass (Da)	Intensity	Calculated Mass (Da)	Identity	Adduct	Error (PPM)
337.1351	14558.6052	337.1046	PA2	M+Na$^+$	90.4699
485.1960	33644.0631	485.1571	PA3	M+Na$^+$	80.2253
633.2572	18847.8522	633.2095	PA4	M+Na$^+$	75.3497
781.3093	7517.3695	781.2619	PA5	M+Na$^+$	60.6034
929.3706	2152.1248	929.3143	PA6	M+Na$^+$	60.5387

Table S5. Peaklist sorted by increasing mass for PA polymer produced from a reaction with a starting PA monomer concentration of 50 mM using CHCA matrix at a constant 500 µL initial reaction volume. Spectrum is shown in Figure S4.

Observed Mass (Da)	Intensity	Calculated Mass (Da)	Identity	Adduct	Error (PPM)

337.1165	19364.2728	337.1046	PA2	M+Na$^+$	35.1570
485.1736	63141.7979	485.1571	PA3	M+Na$^+$	34.1178
633.2316	80578.5785	633.2095	PA4	M+Na$^+$	34.9622
781.2880	72544.4411	781.2619	PA5	M+Na$^+$	33.3382
929.3395	51680.9152	929.3143	PA6	M+Na$^+$	27.1088
1077.3982	32687.4936	1077.3668	PA7	M+Na$^+$	29.1631
1225.4507	18735.4200	1225.4192	PA8	M+Na$^+$	25.6905
1373.5006	9742.7623	1373.4716	PA9	M+Na$^+$	21.1001
1521.5571	4783.5650	1521.5241	PA10	M+Na$^+$	21.7354
1669.6122	2130.5625	1669.5765	PA11	M+Na$^+$	21.3806
1817.6620	1072.3092	1817.6289	PA12	M+Na$^+$	18.1958

Table S6. Peaklist sorted by increasing mass for PA polymer produced from a reaction with a starting PA monomer concentration of 5 mM using CHCA matrix at a constant 500 µL initial reaction volume. Spectrum is shown in Figure S4.

Observed Mass (Da)	Intensity	Calculated Mass (Da)	Identity	Adduct	Error (PPM)
337.1249	4481.2508	337.1046	PA2	M+Na$^+$	60.0406
485.1848	19661.5410	485.1571	PA3	M+Na$^+$	57.2030
633.2380	26464.7655	633.2095	PA4	M+Na$^+$	45.0832
781.2950	24951.3564	781.2619	PA5	M+Na$^+$	42.4006
929.3474	17188.4836	929.3143	PA6	M+Na$^+$	35.5155
1077.4065	11324.2072	1077.3668	PA7	M+Na$^+$	36.8678
1225.4595	6738.4197	1225.4192	PA8	M+Na$^+$	32.8628
1373.5100	3625.6848	1373.4716	PA9	M+Na$^+$	27.9436
1521.5670	1872.4540	1521.5241	PA10	M+Na$^+$	28.2339

Table S7. Peaklist sorted by increasing mass for PA polymer produced from a reaction with a starting PA monomer concentration of 1 mM using CHCA matrix at a constant 500 µL initial reaction volume. Spectrum is shown in Figure S4.

Observed Mass (Da)	Intensity	Calculated Mass (Da)	Identity	Adduct	Error (PPM)
337.1262	1587.7117	337.1046	PA2	$M+Na^+$	63.8837
485.1842	2737.2820	485.1571	PA3	$M+Na^+$	56.0035
633.2412	1356.4569	633.2095	PA4	$M+Na^+$	50.0314
781.2999	419.1161	781.2619	PA5	$M+Na^+$	48.6001
929.3346	127.3205	929.3143	PA6	$M+Na^+$	21.7583

Table S8. Peaklist sorted by increasing mass for PA polymers produced from a reaction with an initial reaction volume of 100 µL using DCTB matrix at a constant 500 mM reactant concentration. Spectrum is shown in Figure 2.

Observed Mass (Da)	Intensity	Calculated Mass (Da)	Identity	Adduct	Error (PPM)
337.1707	751.2521	337.1046	PA2	$M+Na^+$	195.8451
485.2367	7486.8523	485.1571	PA3	$M+Na^+$	164.2001
633.3065	18142.4218	633.2095	PA4	$M+Na^+$	153.2329
781.3729	26575.2316	781.2619	PA5	$M+Na^+$	142.0865
929.4408	24918.1524	929.3143	PA6	$M+Na^+$	136.0680
1077.5071	18015.8145	1077.3668	PA7	$M+Na^+$	130.2306
1225.5658	10376.2752	1225.4192	PA8	$M+Na^+$	119.6276
1373.6302	5075.7174	1373.4716	PA9	$M+Na^+$	115.4877
1521.6912	2261.7859	1521.5241	PA10	$M+Na^+$	109.8268
1669.7599	999.6675	1669.5765	PA11	$M+Na^+$	109.8492

Table S9. Peaklist sorted by increasing mass for PA polymers produced from a reaction with an initial reaction volume of 20 μL using DCTB matrix at a constant 500 mM reactant concentration. Spectrum is shown in Figure 2.

Observed Mass (Da)	Intensity	Calculated Mass (Da)	Identity	Adduct	Error (PPM)
337.1707	809.2554	337.1046	PA2	$M+Na^+$	195.8451
485.2423	8094.6821	485.1571	PA3	$M+Na^+$	175.7113
633.3129	18454.6749	633.2095	PA4	$M+Na^+$	163.3539
781.3729	26472.1600	781.2619	PA5	$M+Na^+$	142.0865
929.4408	25062.2432	929.3143	PA6	$M+Na^+$	136.0680
1077.5071	18371.6887	1077.3668	PA7	$M+Na^+$	130.2306
1225.5658	10627.1711	1225.4192	PA8	$M+Na^+$	119.6276
1373.6302	5710.2410	1373.4716	PA9	$M+Na^+$	115.4877
1521.7010	2536.4816	1521.5241	PA10	$M+Na^+$	116.3253
1669.7599	1171.2960	1669.5765	PA11	$M+Na^+$	109.8492

Table S10. Peaklist sorted by increasing mass for PA polymers produced from a reaction with an initial reaction volume of 5 μL using DCTB matrix at a constant 500 mM reactant concentration. Spectrum is shown in Figure 2.

Observed Mass (Da)	Intensity	Calculated Mass (Da)	Identity	Adduct	Error (PPM)
337.1380	299.9071	337.1046	PA2	$M+Na^+$	98.9796
485.2423	4603.8354	485.1571	PA3	$M+Na^+$	175.7113
633.3129	11558.5177	633.2095	PA4	$M+Na^+$	163.3539
781.3801	15778.2443	781.2619	PA5	$M+Na^+$	151.2270
929.4408	15055.4463	929.3143	PA6	$M+Na^+$	136.0680
1077.5071	11591.1684	1077.3668	PA7	$M+Na^+$	130.2306
1225.5747	7782.5336	1225.4192	PA8	$M+Na^+$	126.8995
1373.6302	4643.7717	1373.4716	PA9	$M+Na^+$	115.4877

1521.7010	2368.9906	1521.5241	PA10	M+Na+	116.3253
1669.7599	1297.7306	1669.5765	PA11	M+Na+	109.8492

Table S11. Peaklist sorted by increasing mass for PA polymers produced from a reaction with an initial reaction volume of 100 µL using CHCA matrix at a constant 500 mM reactant concentration. Spectrum is shown in Figure S7.

Observed Mass (Da)	Intensity	Calculated Mass (Da)	Identity	Adduct	Error (PPM)
337.1603	1098.3100	337.1046	PA2	M+Na+	165.1842
485.2311	8529.8628	485.1571	PA3	M+Na+	152.6890
633.3001	15367.3489	633.2095	PA4	M+Na+	143.1120
781.3658	18175.6860	781.2619	PA5	M+Na+	133.0242
929.4330	16338.5117	929.3143	PA6	M+Na+	127.7269
1077.4987	12284.6729	1077.3668	PA7	M+Na+	122.4126
1225.5658	8120.6730	1225.4192	PA8	M+Na+	119.6276
1373.6302	4877.3538	1373.4716	PA9	M+Na+	115.4877
1521.6912	2550.2962	1521.5241	PA10	M+Na+	109.8268
1669.7495	1257.5810	1669.5765	PA11	M+Na+	103.6345

Table S12. Peaklist sorted by increasing mass for PA polymers produced from a reaction with an initial reaction volume of 20 µL using CHCA matrix at a constant 500 mM reactant concentration. Spectrum is shown in Figure S7.

Observed Mass (Da)	Intensity	Calculated Mass (Da)	Identity	Adduct	Error (PPM)
337.1609	1959.3831	337.1046	PA2	M+Na+	166.8586
485.2311	14503.3107	485.1571	PA3	M+Na+	152.6890
633.3001	24436.8924	633.2095	PA4	M+Na+	143.1120
781.3729	27711.9425	781.2619	PA5	M+Na+	142.0865
929.4330	23660.7712	929.3143	PA6	M+Na+	127.7269

1077.4987	17625.2749	1077.3668	PA7	M+Na$^+$	122.4126
1225.5658	12102.1919	1225.4192	PA8	M+Na$^+$	119.6276
1373.6302	7402.6353	1373.4716	PA9	M+Na$^+$	115.4877
1521.6912	3814.2537	1521.5241	PA10	M+Na$^+$	109.8268
1669.7599	1862.1950	1669.5765	PA11	M+Na$^+$	109.8492

Table S13. Peaklist sorted by increasing mass for PA polymers produced from a reaction with an initial reaction volume of 5 µL using CHCA matrix at a constant 500 mM reactant concentration. Spectrum is shown in Figure S7.

Observed Mass (m/z)	Intensity	Calculated Mass	Identity	Adduct	PPM
337.1661	1183.4624	337.1046	PA2	M+Na$^+$	182.2853
485.2367	7239.8840	485.1571	PA3	M+Na$^+$	164.2001
633.3065	12902.1804	633.2095	PA4	M+Na$^+$	153.2329
781.3729	13419.3787	781.2619	PA5	M+Na$^+$	142.0865
929.4408	12392.0236	929.3143	PA6	M+Na$^+$	136.0680
1077.5071	9747.6587	1077.3668	PA7	M+Na$^+$	130.2306
1225.5747	7213.0047	1225.4192	PA8	M+Na$^+$	126.8995
1373.6396	4544.2198	1373.4716	PA9	M+Na$^+$	122.3312
1521.7010	2385.0717	1521.5241	PA10	M+Na$^+$	116.3253
1669.7599	1341.8829	1669.5765	PA11	M+Na$^+$	109.8492

Table S14. Peaklist sorted by increasing mass for PA polymers produced from a reaction with 1 M NaCl using DCTB matrix at a constant 500 mM reactant concentration and 500 µL reaction volume. Spectrum is shown in Figure 3.

Observed Mass (Da)	Intensity	Calculated Mass (Da)	Identity	Adduct	Error (PPM)
337.0490	1149.2640	337.1046	PA2	M+Na$^+$	165.0015
485.0978	8023.9063	485.1571	PA3	M+Na$^+$	122.1319

633.1415	15451.1507	633.2095	PA4	$M+Na^{+}$	107.4060
781.1852	22824.8838	781.2619	PA5	$M+Na^{+}$	98.1442
929.2332	21926.7933	929.3143	PA6	$M+Na^{+}$	87.3673
1077.2733	18090.3609	1077.3668	PA7	$M+Na^{+}$	86.7472
1225.3157	12981.7564	1225.4192	PA8	$M+Na^{+}$	84.4839
1373.3655	8244.3576	1373.4716	PA9	$M+Na^{+}$	77.2870
1521.4036	4951.2715	1521.5241	PA10	$M+Na^{+}$	79.1927
1669.4502	2415.1078	1669.5765	PA11	$M+Na^{+}$	75.6424
1817.4922	1441.5538	1817.6289	PA12	$M+Na^{+}$	75.2224

Table S15. Peaklist sorted by increasing mass for PA polymers produced from a reaction with 1 M KCl using DCTB matrix at a constant 500 mM reactant concentration and 500 µL reaction volume. Spectrum is shown in Figure 3.

Observed Mass (Da)	Intensity	Calculated Mass (Da)	Identity	Adduct	Error (PPM)
353.0341	2999.3912	353.0786	PA2	$M+K^{+}$	126.0807
501.0808	10505.3693	501.1310	PA3	$M+K^{+}$	100.1564
649.1277	15757.8669	649.1834	PA4	$M+K^{+}$	85.7746
797.1699	16983.7690	797.2359	PA5	$M+K^{+}$	82.7825
945.2169	12013.5540	945.2883	PA6	$M+K^{+}$	75.5622
1093.2618	6870.0096	1093.3407	PA7	$M+K^{+}$	72.1396
1241.3087	3448.5690	1241.3931	PA8	$M+K^{+}$	68.0092
1389.3579	1808.8381	1389.4456	PA9	$M+K^{+}$	63.0898

Table S16. Peaklist sorted by increasing mass for PA polymers produced from a reaction with 1 M NaCl using CHCA matrix at a constant 500 mM reactant concentration and 500 µL reaction volume. Spectrum is shown in Figure S9.

Observed Mass (Da)	Intensity	Calculated Mass (Da)	Identity	Adduct	Error (PPM)
337.1132	2349.0613	337.1046	PA2	$M+Na^+$	25.3811
485.1706	12635.4719	485.1571	PA3	$M+Na^+$	27.8276
633.2246	18340.0201	633.2095	PA4	$M+Na^+$	23.8774
781.2776	17287.7431	781.2619	PA5	$M+Na^+$	20.1353
929.3339	12073.0029	929.3143	PA6	$M+Na^+$	21.0664
1077.3901	7997.3038	1077.3668	PA7	$M+Na^+$	21.6850
1225.4403	4600.6158	1225.4192	PA8	$M+Na^+$	17.2232
1373.4882	2430.9870	1373.4716	PA9	$M+Na^+$	12.0346
1521.5426	1208.7899	1521.5241	PA10	$M+Na^+$	12.1881

Table S17. Peaklist sorted by increasing mass for PA polymers produced from a reaction with 1 M KCl using CHCA matrix at a constant 500 mM reactant concentration and 500 µL reaction volume. Spectrum is shown in Figure S9.

Observed Mass (Da)	Intensity	Calculated Mass (Da)	Identity	Adduct	Error (PPM)
353.0085	2385.0703	353.0786	PA2	$M+K^+$	198.4133
501.0467	9659.9030	501.1310	PA3	$M+K^+$	168.2397
649.0954	18258.6046	649.1834	PA4	$M+K^+$	135.6043
797.1340	22059.8811	797.2359	PA5	$M+K^+$	127.7989
945.1777	16802.7084	945.2883	PA6	$M+K^+$	116.9502
1093.2197	10694.6159	1093.3407	PA7	$M+K^+$	110.6585
1241.2639	5797.3429	1241.3931	PA8	$M+K^+$	104.0975
1389.3104	2685.8536	1389.4456	PA9	$M+K^+$	97.2656
